# Insights into the Degradation of Polymer–Drug Conjugates by an Overexpressed Enzyme in Cancer Cells

**DOI:** 10.1021/acs.jmedchem.2c01781

**Published:** 2023-02-14

**Authors:** Pedro R. Figueiredo, Ricardo D. González, Alexandra T. P. Carvalho

**Affiliations:** † CNC − Center for Neuroscience and Cell Biology, Institute for Interdisciplinary Research (IIIUC), University of Coimbra, 3004-504 Coimbra, Portugal; ‡ PhD Programme in Experimental Biology and Biomedicine, Institute for Interdisciplinary Research (IIIUC), University of Coimbra, Casa Costa Alemão, 3030-789 Coimbra, Portugal; § Department of Biocatalysis and Isotope Chemistry, Almac Sciences, Almac House, 20 Seagoe Industrial Estate, Craigavon BT63 5QD, Northern Ireland, United Kingdom

## Abstract

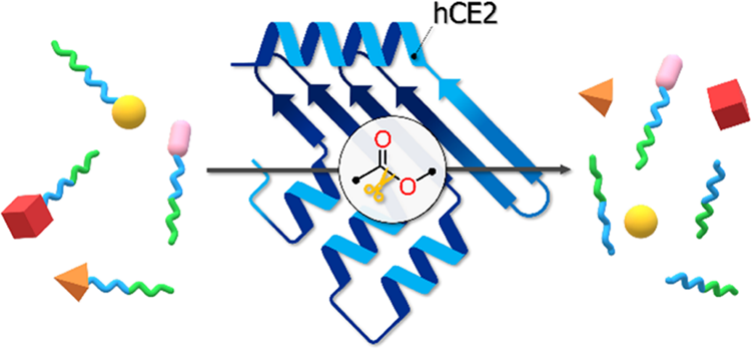

Intensive efforts have been made to provide better treatments to cancer patients. Currently, nanoparticle-based drug delivery systems have gained propulsion, as they can overcome the drawbacks of free drugs. However, drug stability inside the nanocapsule must be ensured to prevent burst release. To overcome this, drugs conjugated to amphiphilic copolymers, assembled into nanoparticles, can provide a sustained release if endogenously degraded. Thus, we have designed and assessed the drug release viability of polymer–drug conjugates by the human Carboxylesterase 2, for a targeted drug activation. We performed molecular dynamics simulations applying a quantum mechanics/molecular mechanics potential to study the degradation profiles of 30 designed conjugates, where six were predicted to be hydrolyzed by this enzyme. We further analyzed the enzyme–substrate environment to delve into what structural features may lead to successful hydrolysis. These findings contribute to the development of new medicines ensuring effective cancer treatments with fewer side effects.

## Introduction

**Figure 1:**
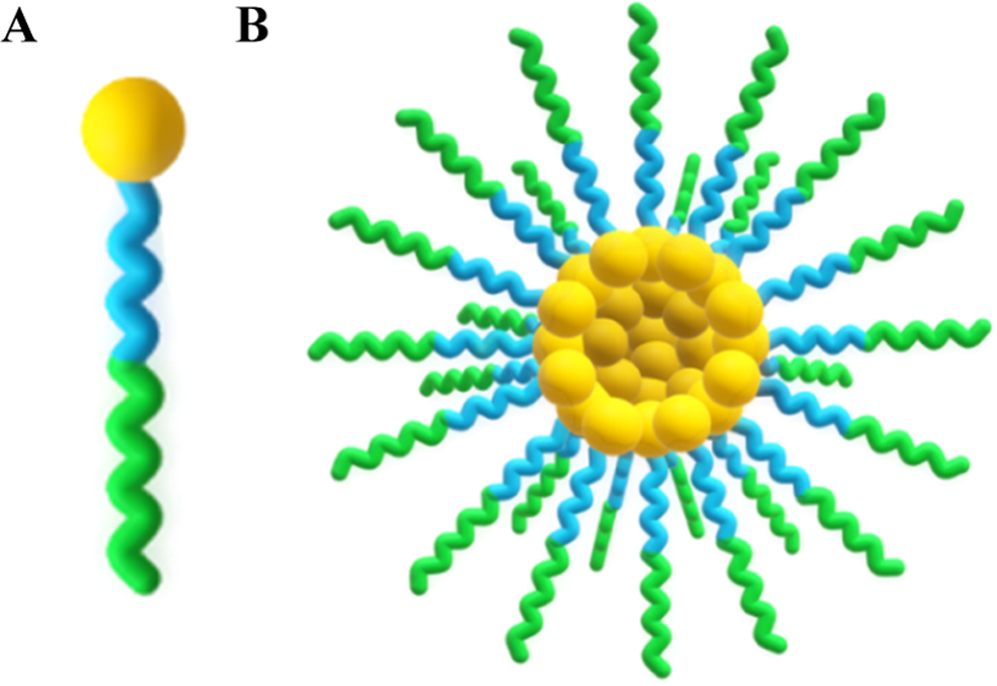
Representation of a PDC (A) and an inverted micelle composed of PDCs (B). Yellow balls represent the anticancer drug that is conjugated with the polymeric carrier (wavy tail). The hydrophobic and hydrophilic units of the polymer are colored blue and green, respectively.

Cancer is a major worldwide problem with 19.3 million new cases estimated in 2020, and its incidence rate is growing along with the longer life expectancy of today’s society.^1^ This leads to the need for better therapies, but, regardless of the huge investments in anticancer therapies, classic chemotherapy still produces the best response rates.^2^ This may be problematic, as free drugs have poor aqueous solubility and short biological half-life, but also because they are often harsh to patients, as they can culminate in high systemic toxicity, and can lead to the development of multidrug resistance, lowering the patients’ life quality.^3^ These limitations have been thoroughly explored, and NP tumor targeting has emerged as a reliable path to overcome them. NPs are widely divided into several categories based on their morphology, size, and chemical properties, which enhances the scope of NPs use.^4^ The drug release from NPs is controlled by several factors, such as drug location, the NP formulation, and the ease with which the drug can diffuse through the matrix system. A major issue with physical encapsulated drugs is the possible occurrence of burst release.^5^ Polymeric NPs, for instance, are nanocapsular shaped, usually organic-based, and are currently being investigated at various phases of clinical trials for advanced delivery, with high interest in the oncological field—breast, colorectal, and non-small-cell lung cancers.^6−8^ Nevertheless, clinical trials of anticancer drugs conjugated to water-soluble polymers were all terminated at different stages due to limited therapeutic response and unexpected toxicity. Part of the failure can be attributed to their small size (<40 kDa), which leads to rapid clearance from circulation through renal filtration.^6^ It is here that the yielding of PDCs (Figure 1A) via conjugation of drugs molecules to bioresorbable polymeric carriers provides plenty of advantages over physical encapsulation and water-soluble polymers. A covalent bond is more effective in stabilizing the drug inside the polymeric micelle and the polymer more resistant to hydrolysis. Also, through PDCs, the drug release inside the tumor tissues can be controlled since the bond between the drug and the polymer needs to be broken to allow drug’s activity. Furthermore, micellar structures of PDCs (Figure 1B) can increase aqueous solubility and stability, extend plasma half-life, and can be engineered for targeted delivery and biodistribution, as well as their size.

In the controlled release of PDCs, the active anticancer drug can be achieved through specific cleavage such that the polymeric units act as a cleavable linker to release the drug from the conjugate. For systemically delivered PDCs, these linkers must be stable in blood circulation to increase drug accumulation in the target sites and to avoid side effects in case of early drug release.

hCE are known to cleave several clinically important classes of drugs (e.g., anticoagulants, angiotensin-converting enzyme inhibitors, antihyperlipidemic agents, antivirals, chemotherapeutics, immunosuppressants, and psychoactive drugs).^9^ To date, six isoforms were identified (hCE1-6) based on sequence homology, where hCE1 and hCE2 are the most relevant and studied isoforms for drug metabolism.^10^ Comparing these isoforms, hCE1 tends to hydrolyze small substrates, esters with small alcohol groups, and large acid groups, whereas hCE2 follows the inverse trend.^11^ As an example, the extensively studied substrate cocaine is metabolized by hCE1 with hydrolysis of methyl ester, while hCE2 catalyzes the cleavage of the benzoyl ester, which is consistent with the structure of the reactive site.^11^

The hCE2 is present within the lumen of the endoplasmic reticulum in many tissues,^12^ and is overexpressed in tumor tissues (in several cancer cell lines), with a small-scale expression in healthy cells. This allows for selective degradation of PDCs in tumor cells^13^ and, therefore, for a targeted action, making it of great interest since it can be used to enhance prodrugs’ activity.^14,15^ Several studies inspected the capacity of this enzyme to hydrolyze chemotherapeutic prodrug agents. A gemcitabine prodrug (LY2334737) was found to be hydrolyzed by hCE2 intracellularly, arresting cell division.^16^ Similarly, a prodrug of 5-fluorouracil (capecitabine), which presents a similar structure to gemcitabine, was found to be cleaved by the enzyme in intestinal tissues, where its expression is relevant, allowing for a better response to the drug in colon cancer patients.^17^ With a more complex structure, three metabolites of the SN38’s prodrug (irinotecan) were found to be metabolized by hCE2 to the drug’s active form.^18^ Following these examples, the enzymatic activation of a doxorubicin derivative prodrug (pentyl PABC–Doxaz) was also explored and demonstrated to be primarily hydrolyzed by the hCE2.^19^ Interestingly, these studies compared the hydrolysis by three hCE isoforms (hCE1-3), and most found that the highest catalytic activity was achieved by hCE2.

In this work, we have designed, tested, and scanned the drug release viability from polymeric models of 30 PDCs through enzymatic degradation using the hCE2. We have evaluated the capacity of hCE2 recognizing the 30 PDCs with covalent docking and MD simulation methods. Then, we applied QM/MM to scan the PES for the acylation (48 trajectories) and deacylation (6 trajectories) steps. For the best-ranked profiles, the FEL were also calculated. We show that drug’s features (smaller size and with a flat core) are key to provide a good binding conformation, which is imperative for the enzymatic hydrolysis to occur.

## Results

### Enzymatic Degradation Mechanism

Previously, we have developed an hCE2 model, as no crystal structure was deposited in the PDB database. We have successfully determined the enzymatic FEL for (−)-cocaine hydrolysis and identified the rate-limiting step as the fourth transition state for this reaction.^20^ The calculated energetic barrier (19.5 kcal mol^–1^) was in excellent agreement with the experimentally determined rate constant (that corresponds to 19.7 kcal mol^–1^).^21^ Also, we showed that the hCE2 active site is located below two mobile α helices (α1 and α10′) that mediate substrate entrance.

**Scheme 1:**
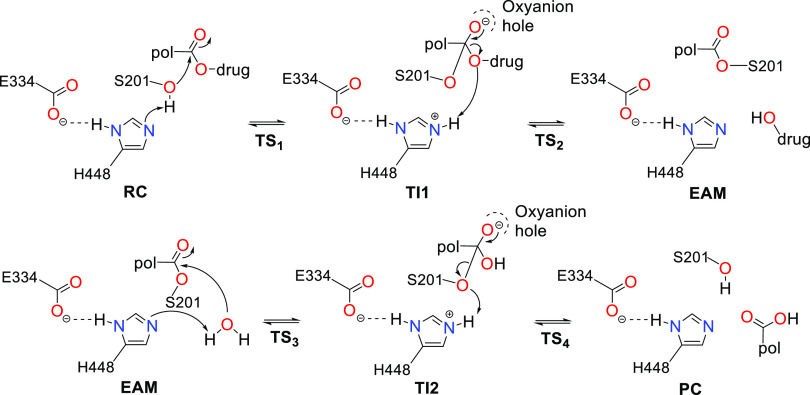
Generalized Enzymatic Degradation Mechanism for the Conversion of PDCs into the Free Drug and Polymeric Carrier The oxyanion hole residues are omitted for simplicity. In the acylation step, the prodrug is activated, and the polymeric carrier is released by the end of the deacylation step.

The active site has two pockets (acyl and alcohol) with different sizes and orientations, and the catalytic triad is composed of S201-H448-E334 and an oxyanion hole region (amide groups of A202, G122, and G123). These amide groups stabilize the negative charge of the tetrahedral intermediates. The S201 residue acts as the nucleophile and the H448 as an acid/base that is stabilized by an E334 residue (Scheme 1). The first part of the PDCs degradation mechanism (usually identified as the acylation step, Scheme 1) concerns the formation of the **EAM** structure, where the active drug is released from the carrier (prodrug activation). The cycle starts with a nucleophilic attack of S201 to the PDC ester group (**RC**) that connects the drug to the polymeric unit.

The hydroxyl hydrogen of S201 is concertedly transferred to the H448 during the **TS1**, to establish the **TI1**. To move the reaction forward, the positively charged histidine needs to transfer the received hydrogen to the drug’s oxygen atom (O_drug_). The negative charge developed in the tetrahedral intermediate oxygen (and stabilized by the oxyanion hole residues) is neutralized with the ester bond breakage (**TS2**) to form the **EAM** structure—and the free drug is released. Then, for the enzyme to activate another drug, the polymeric carrier needs to be released from the enzyme’s active site. This is achieved during deacylation, as it generates the **PC** structure. The nucleophilic attack by a water molecule generates a **TI2**. The water proton is transferred to H448 simultaneously with the nucleophilic attack of the water molecule (through the **TS3**), producing the **TI2**. The polymeric unit is then released from S201 (**PC**) with proton transfer (**TS4**) from H448 to the S201 side-chain oxygen atom (O_S201_).

### Degradation Profile for the Drug Release

The anticancer drugs here explored (gemcitabine, SN38, paclitaxel, and doxorubicin), act by interfering with pathways involved in cancer biology either via DNA modifications, such as phosphorylation or intercalation with the nucleic acids or by inhibiting enzymes important for DNA integrity and structure (e.g., DNA topoisomerases).^23,24^ Some, as irinotecan’s active metabolite SN38 (7-ethyl-10-hydroxycamptothecin) and taxanes (such as paclitaxel), act as co-adjuvant chemotherapy agents of cisplatin or carboplatin, enhancing their activity.^25−27^ These drugs pose clinical importance as they have been shown to be effective in several types of cancers, from (metastatic) non-small-cell lung, pancreatic, bladder, colorectal, ovarian, and breast cancer, with doxorubicin exhibiting a broad activity spectrum.^28^ Nevertheless, side effects have been reported, spanning from dose-related side effects (e.g., doxorubicin can cause nausea, vomiting, myelosuppression, alopecia, and cardiotoxicity),^29^ poor water solubility (taxanes),^30^ cytotoxicity (e.g., SN38 breaks the DNA double-strand),^31^ and systemic effects (e.g., gemcitabine can cause gastrointestinal disturbances, renal impairment, pulmonary toxicity, and influenza-like symptoms).^30^ This leads to the need for careful dose management and prescription according to patients’ needs.

**Figure 2:**
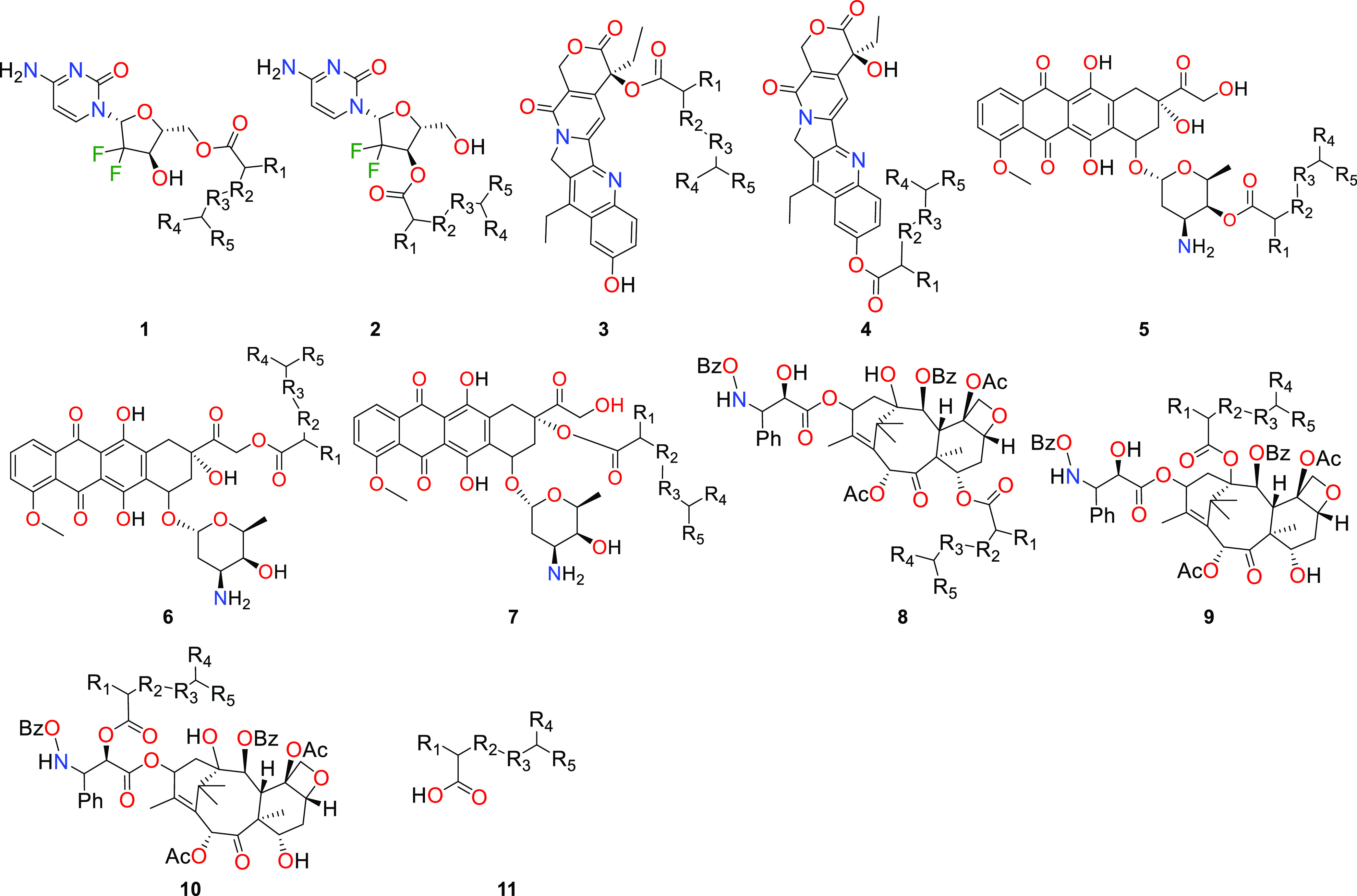
Structure of the designed PDCs based on the gemcitabine (**1-2**), SN38 (**3–4**), doxorubicin (**5–7**), paclitaxel (**8–10**), and polymeric carriers (**11**). **a** (PCL) R_1_,R_4_=H, R_2_,R_3_=CH_2_, R_5_=CH_2_OH; **b** (PGA) R_1_,R_4_=H, R_2_=O, R_3_=CO, R_5_=OH; **c** (PLA) R_1_,R_4_=CH_3_, R_2_=O, R_3_=CO, R_5_=OH.

The designed PDC models (Figure 2) comprise two units: an anticancer drug (gemcitabine, SN38, paclitaxel, or doxorubicin) and a hydrophobic polymeric model (PCL, PGA, and PLA), where the drugs’ free hydroxyl groups were explored for conjugation with the polymeric unit. Concerning the latter, in the PCL derivatives, only one monomer of ε-caprolactone [−C(O)(CH_2_)_5_OH] was modeled, while for the PGA and PLA derivatives, two monomers [−C(O)XOH] (X=CH_2_ for PGA and X=CH_2_(CH_3_) for PLA) were modeled to achieve the same model “size” as PCL. In physiological conditions, these polymeric units are degraded by hydrolysis of their ester linkages. Thanks to their biocompatibility and biodegradability, they are used in biomedical applications such as implants (PCL), suture materials (PGA), and polymeric scaffolds for drug delivery systems (PLA).^22^ To decrease the models’ complexity, we opted not to include the hydrophilic unit (PEG).

**Figure 3:**
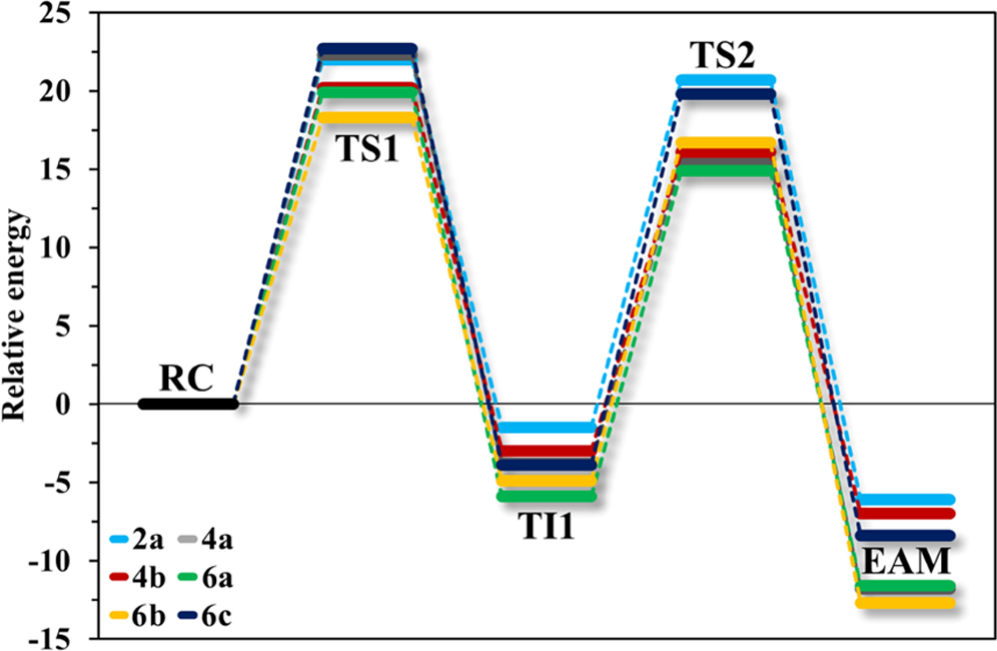
Acylation free energy profiles for the best-ranked PDCs: **2a**, **4a–b**, and **6a–c**, derived from the FEL calculations. The energetic values were calculated with B3LYP-D3/6-31++G(d,p)/MM^32,33^ and are given in kcal mol^–1^: Δ*G*^‡^**TS1** (**2a**:22.0; **4a**:22.3; **4b**:20.2; **6a**:19.9; **6b**:18.3; **6c**:22.7) and Δ*G*^‡^**TS2** (**2a**:22.2; **4a**:19.4; **4b**:19.1; **6a**:20.8; **6b**:21.6; **6c**:23.7). FEL maps and remaining PES profiles are provided in the Supporting Information (Figures S1–S12).

Each considered drug (Figure 2) possesses at least two hydroxyl functions. We have functionalized several of them with a polymeric carrier unit (PCL, PGA, or PLA model). Covalent molecular docking was employed to determine the best **TI1** posing conformation in relation to the hCE2 active site pocket. After MD was applied to the best conformation, the degradation mechanism by the hCE2 was explored with QM/MM simulations with PES scans, from which the best-ranked were further characterized by calculating the FEL (the best-ranked systems are displayed in the manuscript, while the remaining systems are supplied in the Supporting Information with a mechanistic discussion, Figures S1–S12). We considered that free energies above 25.0 kcal mol^–1^ correspond to processes that occur too slowly to be biologically relevant; therefore, we have defined this value as our threshold. The best-ranked systems are summarized in Figure 3, and as discussed later, the polymeric carrier release does not inhibit another enzyme turnover.

We started the screening with the drug molecule depicting the simplest structure (gemcitabine, **1**–**2**) where only two hydroxyl functions are available for conjugation. Then, we increased the systems’ complexity with more hydroxyl functions and molecular size (paclitaxel, **8–10**).

### Gemcitabine-Based Conjugates

**Figure 4:**
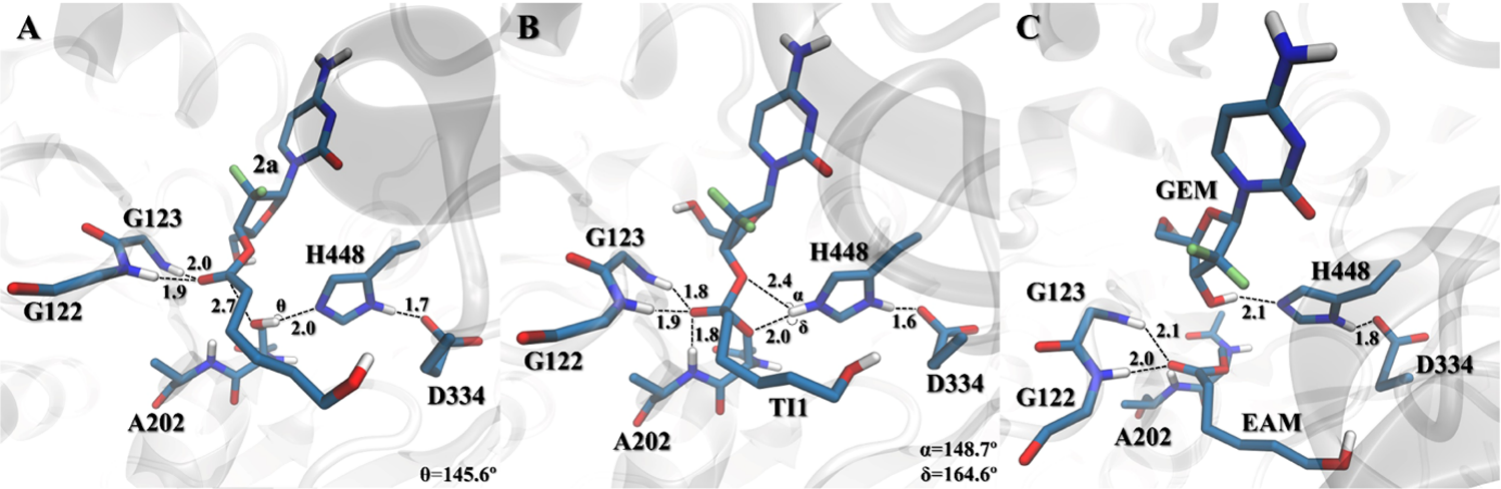
Active site pocket reference structures of the lowest-energy stationary points **RC**, **TI1**, and **EAM** of **2a** (A–C, respectively), where key distances are given in Å, and the free gemcitabine drug is here shortened to **GEM**. Reference structures for **2b–c** are presented in the Supporting Information (Figure S3).

In the gemcitabine conjugates, the drug’s portion at **TI1** interacts with the active site via a hydrogen-bond network between the carbonyl of the drug’s ring, K440 side-chain amine, D447 carboxylate, and the substrate amine with the F81 backbone oxygen. Weak VdW interactions between the L77 and L452 side chains with the substrate aromatic ring were also observed. Contrary to what we have observed for **1a–c**, in **2a–c**, the K448-D477 side chains’ hydrogen bond is not present. Instead, a new NH−π interaction arises between the drug’s amine function and the F81 aromatic ring, and the latter interacts with the L77 side chain via VdW interactions. The Hε_H448_ preference for interaction with the O_S201_ atom at the **TI1** structure is slightly deflected in the case of conjugates **2**, where the Hε_H448_ atom is more positioned in the middle of O_drug_ and O_S201_ (Figures 4 and S2–S4). The energetic barriers for the formation of the **TI1** structure of **1a–c** are always lower than the ones for the drug release (on average less than 8.0 kcal mol^–1^, Figure S1) and well above the defined threshold. This may be correlated with the combination of a higher distance of the O_drug_–Hε_H448_ bond and the angles that are far from the ideal (100.4–111.3°, Figure S2). In opposition, with the functionalization of the other hydroxyl (**2a–c**, Figure 3), the **TI1** formation has an associated barrier lower than drug release. Here, they are always comparable with a small deviation between barriers (on average 0.5 kcal mol^–1^, Figures 3 and S2), showing that the NH−π interaction with F81 could better stabilize the reaction under study.

For the best-ranked conjugate **2a** (Figure 2) the **TS1** has an energetic barrier of 22.0 kcal mol^–1^ (Figures 3 and S1), while the drug release has a similar Δ*G*^‡^ of 22.2 kcal mol^–1^ (Figures 3 and S2). The position of H448 results in a better angle for the forward reaction compared to **2b–c** (Figures 4B and S3B and S3E).

### SN38-Based Conjugates

**Figure 5:**
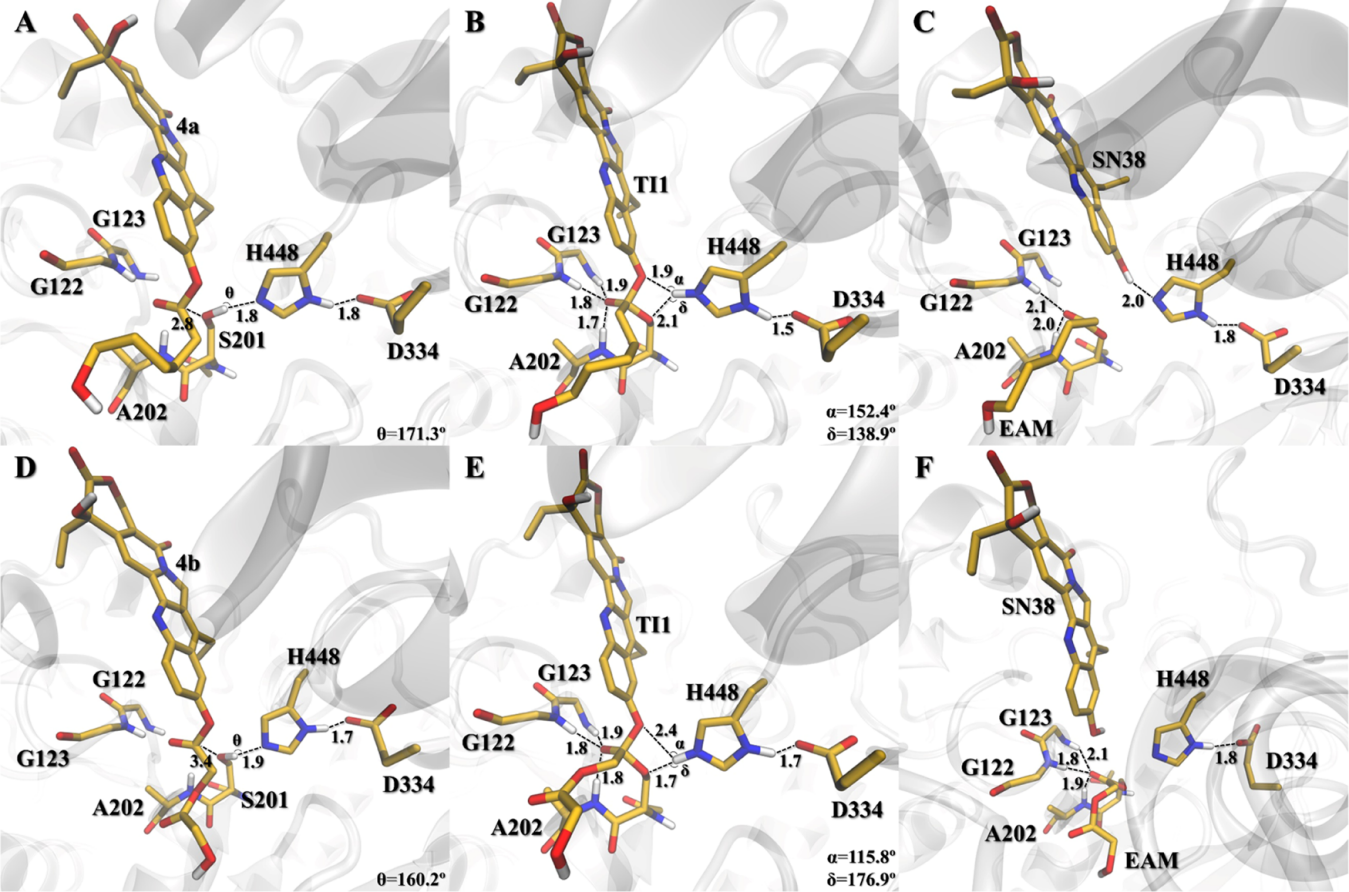
Active site pocket reference structures of the lowest-energy stationary points **RC**, **TI1**, and **EAM** of **4a** (A–C, respectively) and **4b** (D–F, respectively), where key distances are given in Å. Reference structures for **4c** are presented in the Supporting Information (Figure S6).

**Scheme 2:**
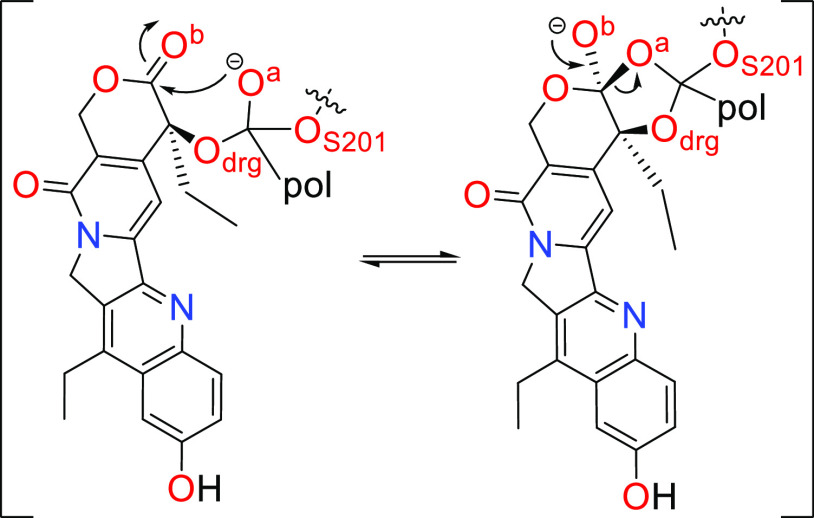
Negative Charge Delocalization of Compounds **3a**–**c TI1** Structure The polymeric unit is here shortened to pol.

In the SN38 conjugates, we observed considerable differences in their free energy barriers (Figure S4). In the allylic derivatives (**3a**–**c**, Figure 2), the calculated Δ*G*^‡^ for **TS1** is 28.5 for **3a**, 22.4 for **3b**, and 16.7 kcal mol^–1^ for **3c** (Figure S4), where the 6.0 kcal mol^–1^ difference between **3b–c** may be attributed to the presence of a methyl group from the PLA unit in the adjacent carbon atom from C_T_, which may assist the formation of the dioxolane ring in the **TI1** structure (explained below). On the benzylic derivatives (**4a–c**, Figure 2), this Δ*G*^‡^ correlates with the angles for the C_T_–O_S201_ bond, with a similar energy value of 21.2 kcal mol^–1^ (Figure S4). In **4c**, a much lower angle (**4c**, Figure S6) induces an increase of 6.0 kcal mol^–1^ in the free energy barrier compared to **4a–b** (Figure 5). When the **TI1** structure of **3a–c** is established (Figures S5B, S5E, and S5H), we observed a peculiar delocalization of the negative charge (Scheme 2) first developed in the carbonyl oxygen atom of the polymer–drug ester group. The negative charge of the O^a^ atom is delocalized between oxygens O^a^ and O^b^ by the formation of a five-membered dioxolane ring (Scheme 2).

The **TI1** conjugated system core interacts with the enzyme mainly by VdW forces and the aromatic hydroxyl function with hydrogen bonds. The L76, L77, L80, and F81 side-chain groups are responsible for the VdW forces in **3a–c**, while fewer were observed in **4a–c**, being mainly performed by A73, L77, and V126 side-chain groups.

On the other hand, the substrate hydroxyl in **3a–c** interacts by hydrogen bonding with the backbone oxygen atom of L80, creating a 4-residue network (L80-S201-K440-D447). In opposition, the portion of the drug in the **4a–c** derivative seems to be able to rotate, as fewer hydrogen bonds are interacting with the substrate core. The majority of the simulated **3** and **4** conjugates (Figures S5 and 5, respectively), have the Hε_H448_ atom closer to the O_drug_ atom than to O_S201_ at the **TI1** structure. Exceptions to this are the **3c** and **4b** that favor the Hε_H448_–O_S201_ bond. The delocalization of the negative charge from O^b^ to O^a^ as the drug is released (**TS2**) in the **3** conjugates requires more than 30.0 kcal mol^–1^ to occur (Figure S4). The fact that the negative charge needs to “move” between more atoms in **3a–c** in contrast to **4a–c** can be one of the possible justifications for the increased amount of energy demanded for the reaction to occur. Accordingly, the Δ*G* toward the **TS2** for **4a–c** equals to around 19.2 kcal mol^–1^ (Figure 3), although a higher barrier was calculated for **4c** (23.1 kcal mol^–1^, Figure S4) that was attributed to the rotation that occurred in the drug’s core, which positioned the reactive atoms in a bad conformation compared to the other **4** derivatives.

### Doxorubicin-Based Conjugates

For the doxorubicin derivatives, we have explored three hydroxyl functions: (i) the hydroxyl of the sugar moiety (**5a–c**), (ii) the one linked to the allylic core ring via the acetyl bridge (**6a–c**), and (iii) the one linked directly to the same ring (**7a–c**, Figure 2). The two hydroxyls from the aromatic core ring (Figure 2) and the amine function were not explored. Due to resonance effects, the hydroxyl proton atoms are placed between the hydroxyl and the nearby carbonyl oxygen atoms (angles for these hydrogen bonds are usually around 150.0° in all of the simulated cases). On the other hand, the functionalization of the amine sugar moiety results in an amide function such that hydrolysis would require a greater energy compared to the ester moiety. As we intend to develop PDCs that could be released *in situ*, we have not explored this position.

The formation of the **TI1** structure for the **5** conjugates is in general higher than our threshold. For **5a**,**c** this value rounds up to 27.0 kcal mol^–1^, while for **5b**, the calculated barrier is less than 4.0 kcal mol^–1^ (Figure S7). Conjugates **6a–c** have a much smaller predicted barrier than **6** and **7** conjugates (being the latter around the threshold limit, Figure S7). This can be related to the drug’s binding position that excels fitting in the hCE2 active site when the acetyl-bridged hydroxyl is functionalized. Consequently, the Δ*G*^‡^ associated with **TS1** for **6a–c** sums to values around 20.5 kcal mol^–1^ (Figure 3). At the intermediate structure, we observed that for **5a–c**, the core rings are mostly exposed to the solvent. Few drug interactions with the protein between the methoxy group and the allylic portion of P441 and M341 lie through VdW. For **6a–c** and **7a–c**, stronger interactions were recorded. The methoxy group is inserted in a small hydrophobic pocket that is composed of A73, L76, L77, and V126 residues. The sugar moiety of **6a–c** interacts weakly with the allylic portion and L452 and via hydrogen bonding with the backbone carbonyl of F81. Additionally, the adjacent hydroxyl interacts with the K440 side chain, which in turn interacts with the carboxylate of D447. Contrarily, the amine function of the sugar moiety of **7a–c** is linked to the backbone carbonyl of H448 via a hydrogen bond, and the hydroxyl weakly with the G449 carbonyl. In **7a–c**, the F81 side chain interacts with the drug with π–π stacking.

**Figure 6:**
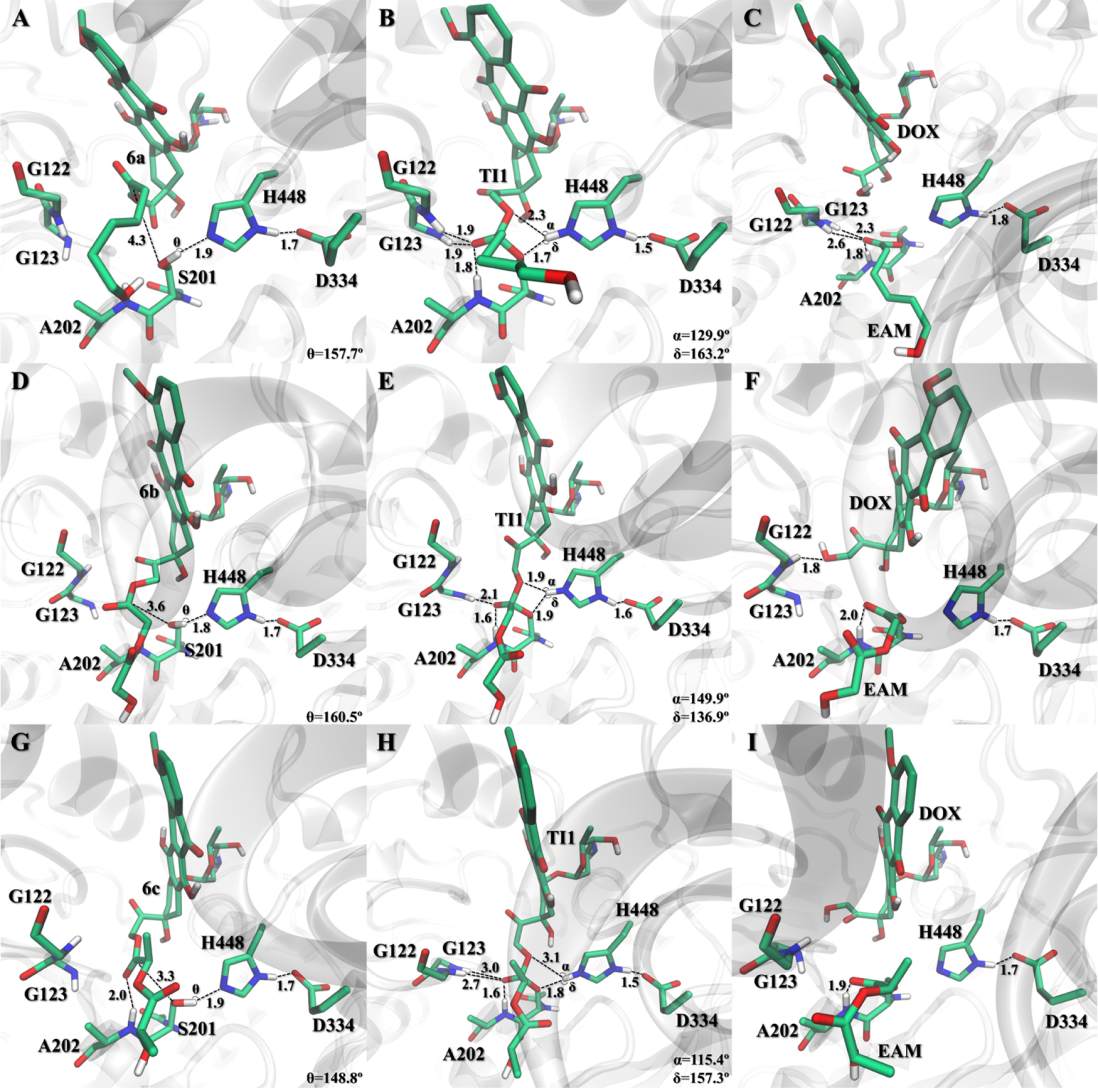
Active site pocket reference structures of the lowest-energy stationary points **RC**, **TI1**, and **EAM** of **6a** (A–C, respectively), **6b** (D–F, respectively), and **6c** (G–I, respectively), where key distances are given in Å, and the free doxorubicin drug here shortened to **DOX**.

The drug release follows the **TS2** achievement. For derivatives **5a–b**, we obtained a value of more than 29.2 kcal mol^–1^, and **5c** requires about 6.0 kcal mol^–1^ less (Figure S7). With the bad positioning of the system (Figure S8), both reactions (**TS1** and **TS2**) are hindered to achieve “efficient” barriers. An example of this is highlighted in derivatives **6a–c** (Figure 6), where lower barriers were calculated, with a ΔG* of around 21.0 kcal mol^–1^ (Figure 3). Meanwhile, for **7a–c**, the **TS2** has a Δ*G*^‡^ of 25.5 kcal mol^–1^ for **7a**. This value increases to 28.0 kcal mol^–1^ in **7b**, and 32.4 kcal mol^–1^ in **7c** (Figure 3).

### Paclitaxel-Based Conjugates

In the most complex systems that we have tested (paclitaxel derivatives, **8**–**10**, Figure 2), the conjugation of bulky groups and their stereo effects prevented a good accommodation of these moieties in the hCE2 active site, as further explained. In the **8** derivatives, the oxyanion hole residues interact weakly with the negatively charged oxygen. This can be explained by stereo effects performed by bulky groups of the drug that perturb the loop containing the oxyanion residues (e.g., the benzamide and phenyl groups near the functionalized hydroxyl, Figures 2 and S12). These disturbances translate into an unstable **TI1** structure that is more than 7.5 kcal mol^–1^ (Figure S10) above the **RC**. In this structure, the PDC’s drug portion interacts strongly with the enzyme via VdW forces carried out by L77, F81, L440, and L452 in the methoxy and methyl groups, and M125, V126, L284, L338, L440, and P441 in the aryl groups. The reaction to **EAM** (Figure S11) requires an enormous amount of energy for the system to reach **TS2** (more than 53.8 kcal mol^–1^, Figure S10). This is related to the higher distance between O_drug_-Hε_H448_ and possibly by stereo effects conducted by the oxetane ring that caps the O_drug_, hindering their approximation to Hε_H448_.

**Figure 7:**
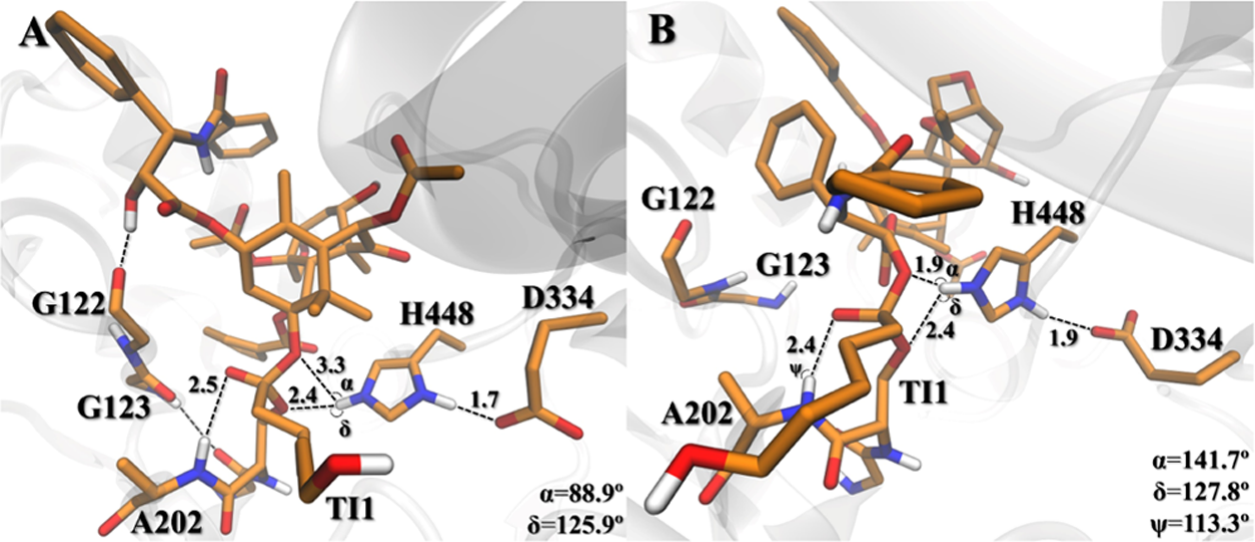
Reference structures of the **TI1** complexes **9a** and **10a** (A and B, respectively) retrieved from the 20 ns aMD last frame run. Distances are given in Å.

For the **9a** and **10a** PDCs, our simulations revealed a distortion of the oxyanion hole loop (Figure 7), where accommodation of these conjugates on the hCE2 active site seems to be problematic. The BzO-NH-Bn set grouped spatially near the functionalized hydroxyl cap (Figures 2 and 7) and perturbed the oxyanion hole loop residues (H120-A128), changing the loop position and inhibiting the interaction of G122 and G123 backbone amides with the negative oxygen atom of the **TI1** (Figure 7). To further evaluate this, we performed 20 ns of aMD to test if there was another enzyme conformation within its energetic landscape that would better accommodate the substrate or a possible reorganization of its tridimensional structure after the conjugates are covalently bonded to the enzyme (in a way that the oxyanion hole loop did not significantly change). In this type of enhanced-sampling method, the energetic barriers that separate different system states are shortened, allowing us to improve the conformational space sampling of the system. This simulation showed that the oxyanion hole loop contains the L124 residue next to the two aromatic groups of the BzO-NH-Bn set (Figure 7). During this run, the average distance between the amide hydrogens of G122 and G123 and the substrate’s negatively charged oxygen was greater than 5.8 Å. The closest distance recorded in the simulated time was also greater than the minimum defined for a hydrogen bond (above 3.4 Å), and the amide backbone of A202 was weakly interacting with the negatively charged substrate’s oxygen. Despite the distance being on average 2.0 Å (showing a possible hydrogen bond), the angle between these atoms thwarting the hydrogen interaction.

Although we were able to scan the drug release reaction for **8a–c**, the associated energy barriers hinder their catalysis, as the oxyanion hole residues are weakly stabilizing the negative charge for all tested paclitaxel-based conjugates. The oxyanion hole promotes catalysis with the stabilization of the negative charge developed in the tetrahedral intermediates; without this stabilization, the activation energy required would be exorbitant, therefore inhibiting the catalytic reaction.^34^ Moreover, the position of the catalytic histidine may play an important role in lowering the energetic barrier as seen for other enzymes and different substrates.^35,36^ Therefore, we predict that these conjugates (**8**–**10**, Figure 2) may be hardly hydrolyzed by the hCE2 due to a high energetic barrier.

### Polymeric Carrier Release

The recycling of the enzyme for another PDC activation reaction requires the removal of the polymeric carrier (**11**, Figure 2), which was left covalently bonded to the S201 side chain after the drug release (Scheme 1). The resulting biocompatible and biodegradable polymeric product will then be excreted from the body.^37^

**Figure 8:**
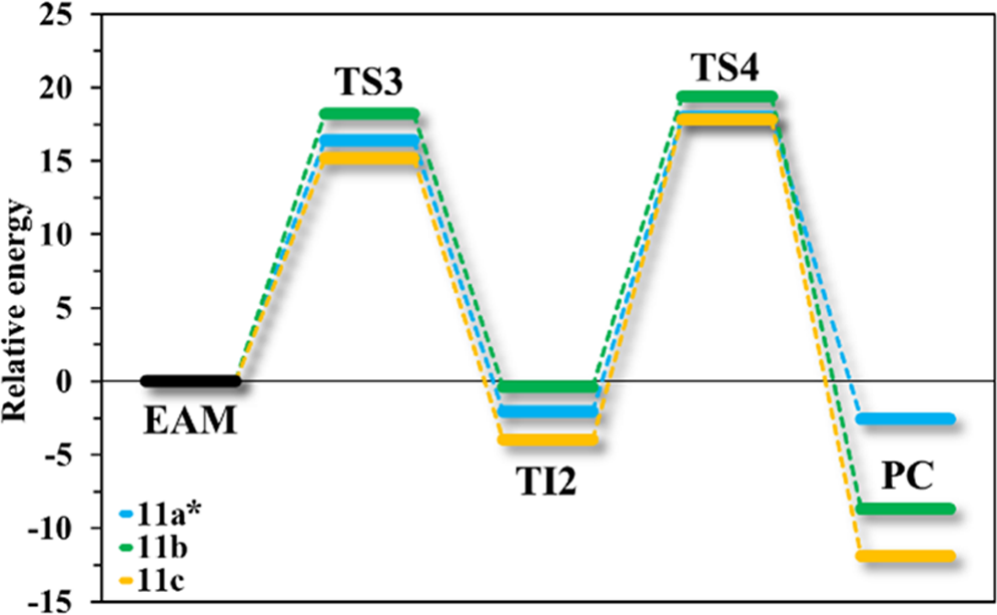
Free energy profiles for the deacylation step of carriers **11b**–**c**, derived from the PES, and for **11a** from the *FEL. Relative barriers are given in kcal mol^–1^ for **TS3** (**11a**:16.4*, **11b**:18.2, **11c**:15.2) and **TS4** (**11a**:20.1*, **11b**:19.8, **11c**:21.8). The FEL maps for **11a** are presented in the Supporting Information (Figure S12).

**Figure 9:**
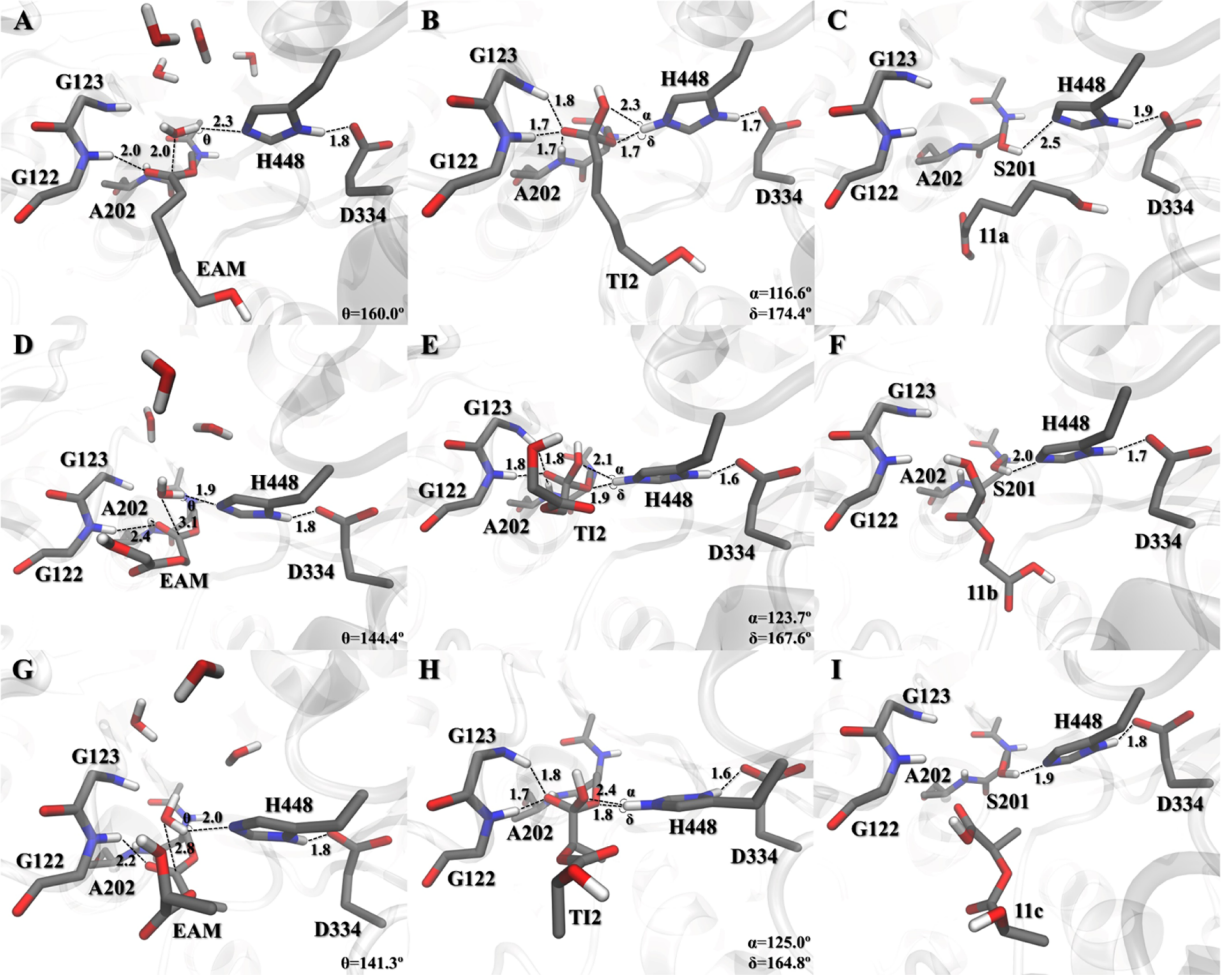
Active site pocket reference structures of the lowest-energy stationary points **EAM**, **TI2**, and **PC** of **11a** (**A**, **B**, and **C**, respectively), **11b** (**D**, **E**, and **F**, respectively), and **11c** (**G**, **H**, and **I**, respectively), where key distances are given in Å.

Several water molecules are able to access the hCE2 active site and are responsible for moving the reaction forward. The Δ*G*^‡^ for the first step of polymer release (**TS3**) ranges from 15.2 to 18.2 kcal mol^–1^ (Figure 8). After the nucleophilic attack and hydrogen transfer to the Nε_H448_ atom, the **TI2** complex is generated (Figure 9B,E,H). The backbone amides of G122, G123, and A202 are stabilizing the negative charge developed with the nucleophilic attack. The **TI2** polymeric chain interacts mostly with hydrophobic residues since the acyl pocket has a more hydrophobic environment (composed mostly of leucine residues) compared to the alcohol pocket.^16^ On the other hand, the **TI2** hydroxyl unit does not interact with any residue (aside from H448) once it is pointing toward the active site exit. In this structure, Hε_H448_ shows a preference for interaction with the O_S201_ atom compared to O_WAT_ (Figure 9B,E,H). This may reflect the stabilization of the **TI2** structure, as the backward reaction (toward the **TS3**) is, in terms of energy, less costly than moving forward to the products (through **TS4**, Figure 8). This may also be the reason why **11c** has the highest energetic barrier to **TS4** (Δ*G*^‡^ of 21.8 kcal mol^–1^). Nevertheless, in all of the cases, the **TS4** relative energy is similar with an average ΔG* of 20.5 kcal mol^–1^ (Figure 8). Finally, the polymeric model (deacylation product) is released from the enzyme after the last transition state (**TS4**) to yield the **PC**. After this point, the polymeric carrier is released from the hCE2 active site, excreted from the body, and the catalyst is ready for another turnover. The deacylation rate-limiting step is comparable in all three cases (19.8–21.8 kcal mol^–1^ for **11a**–**c**, Figure 8) showing that the polymer elimination from the hCE2 active site does not compromise the continuity of PDC degradation.

## Conclusions

We have screened the hCE2 capability to accept and degrade 30 PDCs by means of covalent molecular docking, molecular dynamics, and QM/MM MD calculations. The formation of PDC micellar structures (Figure 1B) can enhance plasma stability and ultimately achieve accumulation in the tumor site. As the hCE2 is overexpressed in tumor cells, selective degradation of those conjugates (prodrug activation) inside them could be expected.

The screened conjugates are composed of an anticancer drug covalently linked to a polymeric carrier through an ester bond. We have tested several hydroxyl functions of each drug to evaluate differences in the free energy barriers for the drug and polymeric carrier release. As an initial screening, we have performed PES scans on the whole reaction mechanism cycle to assess the associated barriers (acylation step: formation of **TI1** and **EAM** structures; deacylation step: formation of **TI2** and **PC** structures, Scheme 1). The most feasible reactions were further characterized by calculating the FEL for those systems, where the rate-limiting step is under the 25.0 kcal mol^–1^ threshold (Figure 2) and associated with the drug’s release. For the acylation step of the best-ranked systems (**2a**, **4a–b**, and **9a–c**), we predicted a Δ*G*^‡^ below 23.7 kcal mol^–1^, where the best-ranked conjugate (**4b**) has a Δ*G*^‡^ of 20.2 kcal mol^–1^.

Based on the results of this screening, some key aspects can be retrieved to understand the feasibility of designed PDCs capable to suffer hydrolysis by the hCE2: the drug’s size in conjugation with a good binding spatial conformation seems to be fundamental. For example, we have captured a gemcitabine candidate (**2a**, Figure 4) whereas, in all tested paclitaxel conjugates, none were able to achieve an energetic barrier below the defined threshold. In this latter case, it was due to a perturbation of the oxyanion hole that is essential for adequate stabilization of the intermediate’s negative charge (Scheme 1). The binding positioning is also displayed on the SN38 (**4a–b**, Figure 5) and doxorubicin (**6a–c**, Figure 6) conjugates. As these drugs display a flat core, binding to the hCE2 pocket seems to be facilitated. For the SN38, and in addition to the previous factor, the formation of a secondary structure that implies a “one-way” movement of the negative charge (Scheme 2), sounds for higher energetic barriers as the restoration of the carbonyl function may assist bond breakage. Here, we observed the contrary: bond breakage in the **3** derivatives may assist the movement of the negative charge to the primary oxygen atom (O^a^, Scheme 2), so the carbonyl can be restored.

Finally, and to ensure that the enzyme was not covalently blocked by the leaving polymeric carrier after drug release, we have also investigated the deacylation phase. We observed a Δ*G*^‡^ below 21.8 kcal mol^–1^ associated with the last transition state for the simulated polymeric models, showing the possibility for another turnover in PDC hydrolysis.

Several works delved into the expression of hCE2 in different tissues, and its overexpression in tumor cells.^38−40^ By relying on this particular pattern, advanced therapies and delivery systems can use this advantage for better treatment options. Here we have explored this with an array of small drugs already in use for a range of cancer types, enhancing their scope. The engineering of polymeric micelles for their delivery diminishes the probability of off-target effects, which are major drawbacks of current drug therapies. Furthermore, the activation of those (pro)drugs only upon tumor cell entry and localization allows for an increased therapeutic effect with smaller doses, preventing the need for large amounts of raw materials for the fabrication of these medicines by industries, while allowing patients to develop fewer side effects and enhancing drugs’ effectiveness. By expanding the scope of viable PDCs, we are contributing to the solution of one of the most urgent health issues of today’s society that is to ensure effective cancer treatments while lowering side effects that are a consequence of the administration mode, formulation, and status of the drugs, self-guarding healthy lives and promoting well-being for all.

## Methods

### Initial Setup

The hCE2 structure was previously modeled and reported by our group, where detailed information regarding the modeling procedure can be found.^20^ The model was built using Modeller v9.24^41^ followed by cMD (100 ns) prior to aMD (50 ns) to obtain the enzyme’s active form. Our model is in high agreement with the one concomitantly reported in the α Fold database,^42^ with only 0.3 Å of RMSD (Cα, excluding the N- and C- terminal ends).^20^ The protonation state residues of the hCE2 were assigned considering an aqueous solution with physiological pH (7.4).^43^ The **TI1** and **TI2** residues were geometry-optimized in Gaussian09^44^ using B3LYP^32^ with the 6-31G(d,p) basis set in the gas phase. The atomic partial charges were calculated for **TI1** and **TI2** according to the RESP methodology.^45,46^

### Molecular Docking

The initial position of the **TI1** structures was obtained by the flexible side-chain covalent docking method,^47^ performed with the AutoDock4.2 suite of programs, using the LGA.^48^ A total of 150 runs were carried out. The population was set to 300, the maximum number of generations to 27,000, and the maximum number of energy evaluations to 2,500,000. The best-ranked result was selected, where the polymeric unit and drug are placed in the correct pocket and subjected to MD simulations.

### Molecular Dynamics

The cMD and aMD simulations were performed using the AMBER18^49^ with the parm99SB^50^ and GAFF^51^ force fields. The structures were placed within a preequilibrated octahedral box of TIP3P waters^52^ (10.0 Å distance between the surface of the protein to the box) with 6 Na^+^ ions to neutralize the system. Two initial energy minimizations and 500 ps of equilibration were carried out in an NVT ensemble using Langevin dynamics with small restraints of 10.0 kcal mol^–1^ on the protein, to heat the systems; 5 ns production simulations were carried out at 310.15 K in an NPT ensemble using the Langevin dynamics with a collision frequency of 1 ps^–1^. Constant pressure periodic boundary conditions were imposed with an average pressure of 1 atm. Isotropic position scaling was used to maintain pressure with a relaxation time of 2 ps, and the time step was set to 2 fs. SHAKE constraints were applied to all bonds involving hydrogen atoms.^53^ The PME method^54^ was used to calculate electrostatic interactions with a cutoff distance of 10.0 Å. The aMD simulations were conducted for 20 ns per complex and the parameters were selected based on the system size, average dihedral, and potential energy.^55^ These parameters are provided in Table S1.

### Quantum Mechanics/Molecular Mechanics Calculations

The QM/MM Umbrella Sampling calculations^56−58^ were performed using the internal semiempirical hybrid QM/MM functionality implemented in AMBER18^49^ with periodic boundary conditions. The last cMD reference structure of each complex was used as the initial structure for the QM/MM calculations. The PM3 semiempirical method^59,60^ was employed to describe the QM region, while the MM region was described by the Amber parm99SB force field.^50^ Reactions were conducted at physiological temperature (310.15 K). Electrostatic embedding^61^ was employed and the boundary was treated via the link atom approach. Long-range electrostatic interactions were described by an adapted implementation of the PME method^54^ for QM/MM.^62^

For the PES and FEL scans, the same combination of atoms representing the reaction coordinates was used (Figures S13 and S14): (i) the distance between Hε_H448_–O_S201_ (θ) connected to O_S201_–C_T_ (ω) and (ii) the distance between Hε_H448_–O_drug_ (ϕ) connected to O_drug_–C_T_ (σ). In PES, the reaction coordinates (distance between Hε_H448_ and O_S201_ (θ) or O_drug_ (ϕ), Figure S13) were restrained in 0.1 Å steps using the umbrella sampling method, except near the transition states where smaller 0.02 Å steps were employed, with an umbrella constraint force of 200 kJ mol^–1^ A^–2^. For every window, a total of 50 ps were simulated with a time step of 1 fs. For the FEL, and in accordance with our previous work,^20,35^ the structures from PES were used as starting points. To keep the reaction coordinates at the requested distances and to ensure enough overlap between windows, an umbrella constraint force of 200 kJ mol^–1^ A^–2^ was used along the reaction path and incrementally increased to 1000 kJ mol^–1^ A^–2^ as the reaction coordinates deflect from the minimum-energy path. For every window, a total of 20 ps was simulated with a time step of 1 fs and the distances scanned from 1.4 to 2.5 Å (ω and σ, Figure S13) and from 1.0 to 2.0 Å (θ and ϕ, Figure S13), comprising a total of 132 simulations per step. The free energy profiles were obtained by combining all of the statistics from each reaction simulation by resorting to WHAM with the Monte Carlo bootstrap error analysis.^63,64^ The minimum-energy path was traced with the MEPSA software v1.4,^65^ and all of the averaged minima structures were retrieved and optimized with PM6/parm99SB to make all of the figures.

### Density Functional Theory Corrections

For the high-level layer corrections, we resorted to a method described in ref (66), which is based on the work of Truhlar and co-workers.^67,68^ This method compensates for the limitations of semiempirical methods. Multiple structures of the QM region (with H link atoms) were extracted and submitted to single-point energy calculations in the gas phase. Structures were extracted along the reaction path (for PES) and from the whole potential of mean force (for FEL). The calculations were carried out in ORCA software v5.0.1,^69^ with the semiempirical PM3^59,60^ and B3LYP-D3/6-31++G(d,p).^32,33^ The corrected energy term (*E*_corr_) was interpolated from those structures employing eq 1
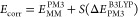
1where the term Δ*E*_PM3_^B3LYP^ corresponds to the difference between the free energy for the high-level layer set, calculated with B3LYP-D3/6-31++G(d,p)^32,33^ and PM3,^59,60^ while *S* represents the cubic spline function of the difference between the high-level and low-level theories representing the QM region.

## Supporting Information Available

PES profiles for PDCs **1a–c** and **2b–c** (Figure S1), **3a–c** and **4b–c** (Figure S2), **5a–c**, **6a–c**, and **7a–c** (Figure S7), **8b–c** (Figure S10); FEL maps for PDCs **2a** (Figure S1), **4a–b** (Figure S4), **6a–c** (Figure S7), **11a** (Figure S12); active site reference structures for the stationary points of **1a–c** (Figure S2), **2b–c** (Figure S3), **3a–c** (Figure S5), **4c** (Figure S6), **5a–c** (Figure S8), **7a–c** (Figure S9), **8b–c** (Figure S11); aMD parameters (Table S1); general QM region selected (Figure S13); and full representation of the selected QM region (Figure S14) (PDF)
PDB database (ZIP)

## Abbreviations Used

AMBER18: Amber molecular dynamics program
aMD: accelerated molecular dynamics
cMD: conventional molecular dynamics
EAM: enzyme-acyl monomer
FES: free energy surface
GAFF: general Amber force field
hCE: human carboxylesterase
HF: Hartree–Fock
LGA: Lamarckian genetic algorithm
MD: molecular dynamics
MM: molecular mechanics
NP: nanoparticle
PC: product complex
PCL: poly(caprolactone)
PDC: polymer–drug conjugates
PES: potential energy surface
PGA: poly(glycolic acid)
PLA: poly(lactic acid)
PME: particle mesh Ewald
QM: quantum mechanics
QM/MM: quantum mechanics/molecular mechanics
RC: reactant complex
RESP: restrained electrostatic potential
TI: tetrahedral Intermediate
TS: transition state
VdW: Van der Waals
WHAM: weighted histogram analysis method
