# Supplementary material for: Insights into the Degradation of Polymer–Drug Conjugates by an Overexpressed Enzyme in Cancer Cells
Source: J Med Chem. 2023 Feb 14;66(4):2761–72. doi: 10.1021/acs.jmedchem.2c01781 (PMC9969400; doi:10.1021/acs.jmedchem.2c01781)
Supplement: Supplementary file 1 — jm2c01781_si_001.pdf [file jm2c01781_si_001.pdf]

Supporting Information

Insights in the Degradation of Polymer-Drug Conjugates by an Overexpressed Enzyme in Cancer Cells

Pedro R. Figueiredo,[δξ] *Ricardo D. González,*[δξ] *and Alexandra T.P. Carvalho*[δϕ*]

[δ] CNC – Center for Neuroscience and Cell Biology, Institute for Interdisciplinary Research (IIIUC), University of Coimbra, 3004-504 Coimbra, Portugal

[ξ] PhD Programme in Experimental Biology and Biomedicine, Institute for Interdisciplinary Research (IIIUC), University of Coimbra, Casa Costa Alemão, 3030-789 Coimbra, Portugal

[ϕ] Almac Sciences, Department of Biocatalysis and Isotope Chemistry, Almac House, 20 Seagoe Industrial Estate, Craigavon, BT63 5QD, Northern Ireland, United Kingdom

[*] atpcarvalho@uc.pt

Table of Contents

Figure S1. Acylation profiles of PDCs **1a-c** and **2b-c**, and FEL maps of **2a.** S2

Figure S2. Active site pocket reference structures of **1a-c**. S2

Figure S3. Active site pocket reference structures of **2b-c**. S3

Figure S4. Acylation profiles of PDCs **3a-c** and **4b-c**, and FEL maps of **4a-b**. S4

Figure S5. Active site pocket reference structures of **3a-c**. S5

Figure S6. Active site pocket reference structures of **4c**. S5

Figure S7. Acylation profiles of PDCs **5a-c**, **6a-c**, and **7b-c**, and FEL maps of **6a-c**. S6

Figure S8. Active site pocket reference structures of **5a-c**. S7

Figure S9. Active site pocket reference structures of **7b-c**. S8

Figure S10. Acylation profiles of PDCs **8a-c**. S9

Figure S11. Active site pocket reference structures of **8a-c**. S10

Figure S12. Deacylation step FEL maps of **11a**. S11

Table S1. aMD simulations parameters. S11

Figure S13. General QM region selected. S11

Figure S14. Representation of the complete QM region selected for the **TI1** and **TI2** S12

Gemcitabine-based conjugates

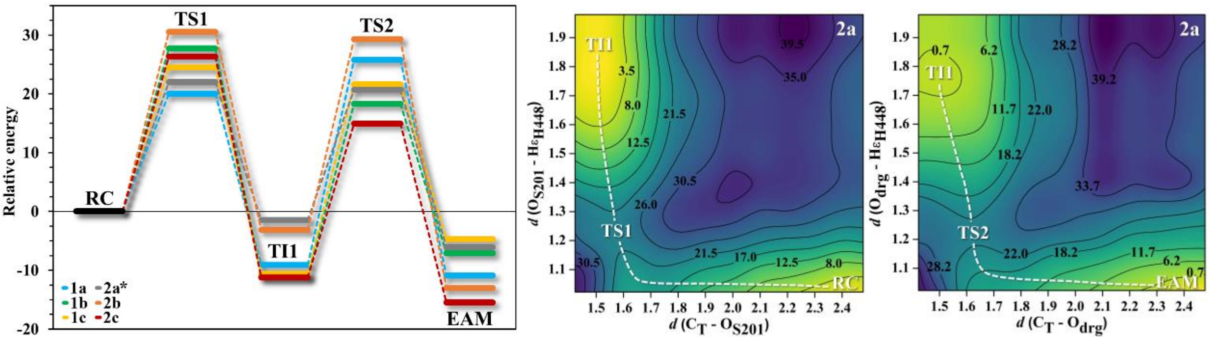

Figure S1. Free energy profiles of PDCs **1a-c** and **2b-c** (**left**) and FEL maps of **2a** (**right**) for the acylation step derived from the PES and *FEL. The energetic values were calculated with B3LYP-D3/6-31++G(d,p)/MM[25,51] and are given in kcal mol^{-1}: $\Delta G^{\ddagger}$ **TS1** (**1a**:20.0; **1b**:27.7; **1c**:24.5; **2a**:22.0*; **2b**:30.5; **2c**:26.3) and $\Delta G^{\ddagger}$ **TS2** (**1a**:34.9; **1b**:29.3; **1c**:32.2; **2a**:22.2*; **2b**:29.3; **2c**:26.1).

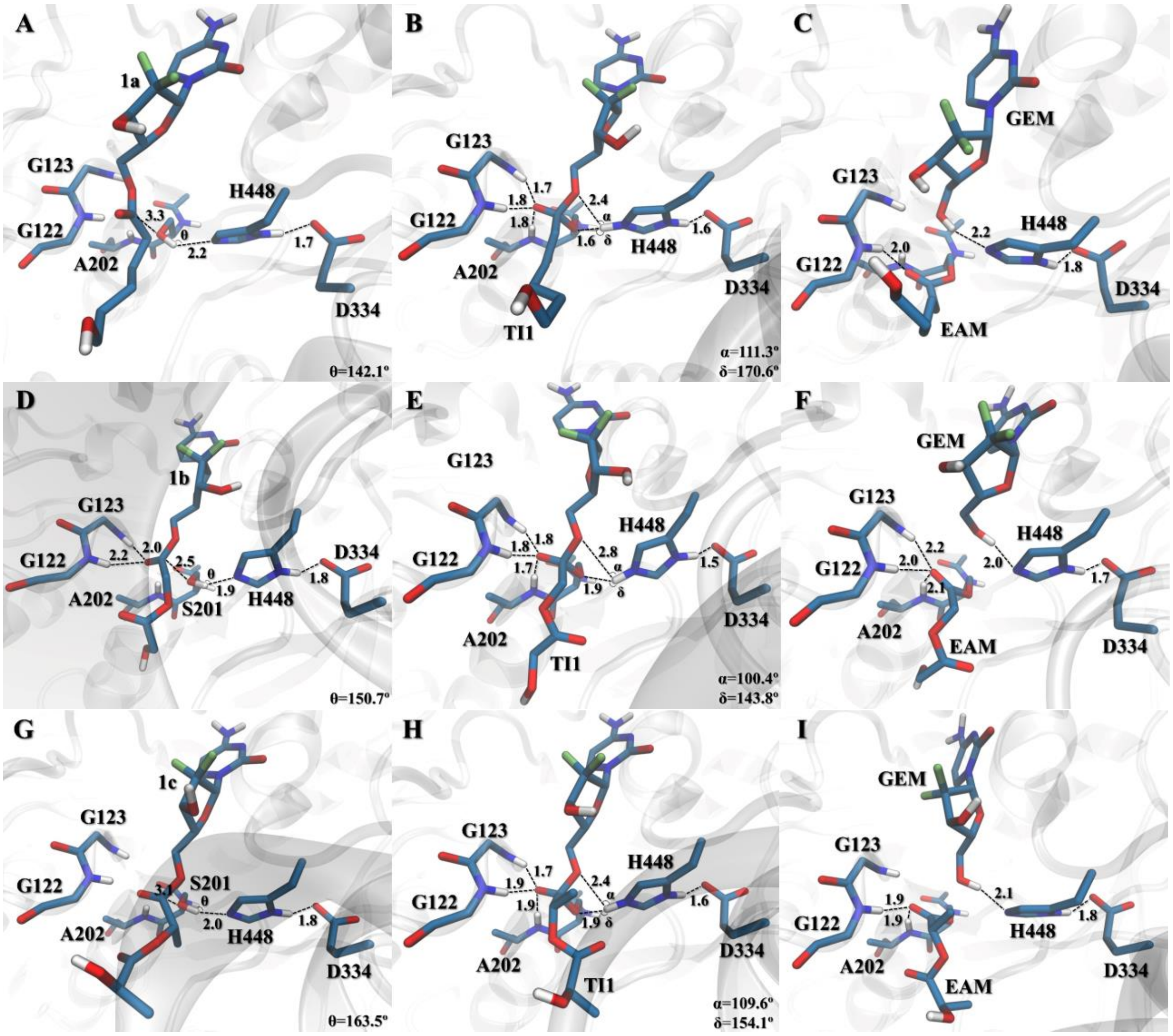

Figure S2. Active site pocket reference structures of the lowest energy stationary points **RC**, **TI1,** and **EAM** of **1a** (**A**, **B**, and **C**, respectively), **1b** (**D**, **E**, and **F**, respectively), and **1c** (**G**, **H**, and **I**, respectively), where key distances are given in Å, and the free gemcitabine drug here shortened to **GEM**.

Starting with derivatives **1a-c**, the **RC** structures have the substrate C_T atom below 3.3 Å from the O_{S201}, where the corresponding hydrogen is below 2.2 Å from the $N\varepsilon_{H448}$ (Figures S3A, S3D, and S3G). The angles are aligned to move the reaction to **TS1**, with the worst result for **1a** (140.1°, Figure S3A). The free energy barriers for the forward reaction deviate from 20.0 to 27.7 kcal mol^{-1} (Figures S2). The **TI1** is 29.0 kcal mol^{-1} exergonic in relation to the **RC** (Figure S2) and in this latter structure, all three hydrogen bonds of the oxyanion hole are stabilizing the negative charge (Figures S3B, S3E, and S3H). The reaction evolves to the **EAM** through the **TS2**, which has a $\Delta G^{\ddagger}$ 29.3, 32.2, and 34.9 kcal mol^{-1} for **1a-c**, respectively (Figure S2). After drug release (**EAM**, Figures S3C, S3F, and S3I), the newly generated hydroxyl interacts with the $N\varepsilon_{H448}$, as the drug leaves the active site, and the polymeric chain attached to S201 keeps interacting with the oxyanion hole residues (even though already in the form of carbonyl).

Then, we screened the other derivatives (**2a-c**, Figure 3). These have the functionalized hydroxyl directly linked to the oxolane ring. These **RC** structures are in general below 3.0 Å from S201 and, as the substrate prepares to react with the enzyme, the oxyanion hole residues start to interact with the oxygen atom that will develop the negative charge (Figures 5, S4A, and S4D). Proton transfer from S201 to H448 will occur with the **TS1** that is located at 22.0 and 26.3 kcal mol^{-1} for **2a** and **2c**, respectively, while **2b** is located slightly higher (30.5 kcal mol^{-1}, Figure S2). Moving to the **TS2**, the intermediate **2a** has a $\Delta G^{\ddagger}$ of 22.2 kcal mol^{-1}, while in **2b-c** this barrier amounts to 29.3 and 26.1 kcal mol^{-1}, respectively (Figure S2). The acylation step ends with the drug release (**EAM**, Figures 5C, S4C, and S4F), which has lower energy than that of **TI1**. Alike the **1a-c**, the released gemcitabine drug interacts with H448 as it moves out of the reactive center. From these gemcitabine-based conjugates, we have calculated one with a barrier below our threshold (**2a**) with 24.8 kcal mol^{-1} for the **TS2** in the PES (Figure S2). The FEL calculations for this conjugate showed a decrease in $\Delta G^{\ddagger}$ of 2.8 kcal mol^{-1} and similar energy for the **TS1** in both PES and FEL calculations (21.9 *versus* 22.0 kcal mol^{-1}, Figure S2). Considering the FEL for **2a** and PES for **2b-c**, the $\Delta G^{\ddagger}$ are similar in all three cases (Figure S2).

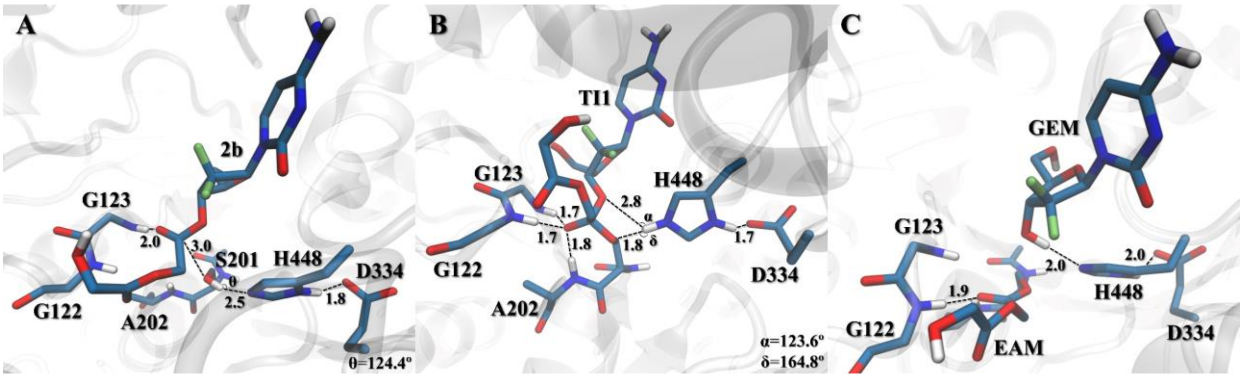

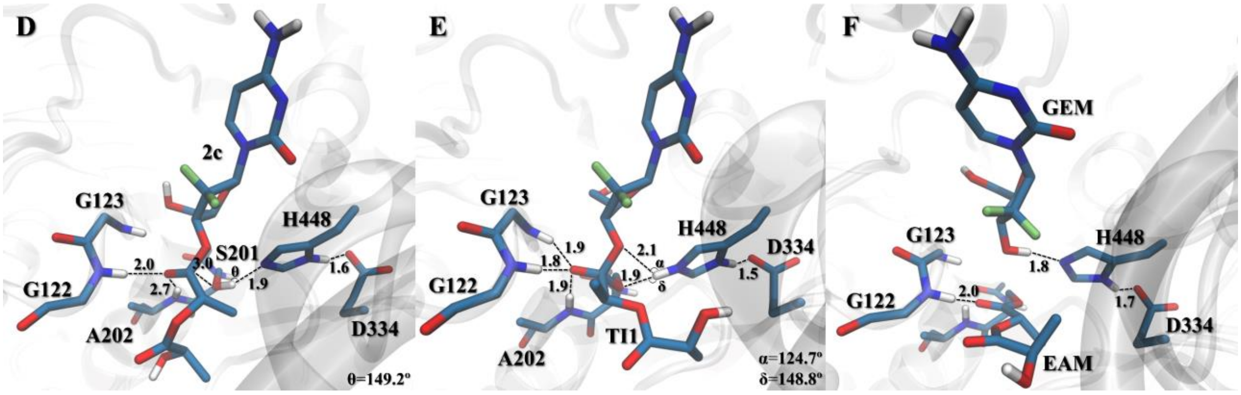

Figure S3. Active site pocket reference structures of the lowest energy stationary points **RC**, **TI1**, and **EAM** of **2b** (**A**, **B**, and **C**, respectively) and **2c** (**D**, **E**, and **F**, respectively), where key distances are given in Å, and the free gemcitabine drug shortened to **GEM**.

SN38-based conjugates

For the allylic derivatives (**3a-c**) in the **RC** (Figures S6A, S6D, and S6G), the carbonyl oxygen atom of the drug's dihydropyran ring is pointing to the amide groups of G122, G123, and A202 oxyanion hole residues. This may be responsible for placing the **3a-c** carbonyl from the ester group in a favorable position for nucleophilic attack. The distance from the S201 hydroxyl hydrogen to the H448 rounds 2.2 Å with angles above 158.0°, while the carbonyl carbon atom that is going to be attacked and the S201 hydroxyl oxygen are in general above 3.0 Å apart. After the nucleophilic attack, the **TI1** is established and located more than 5.4 kcal mol^{-1} below the **RC** (Figure S5).

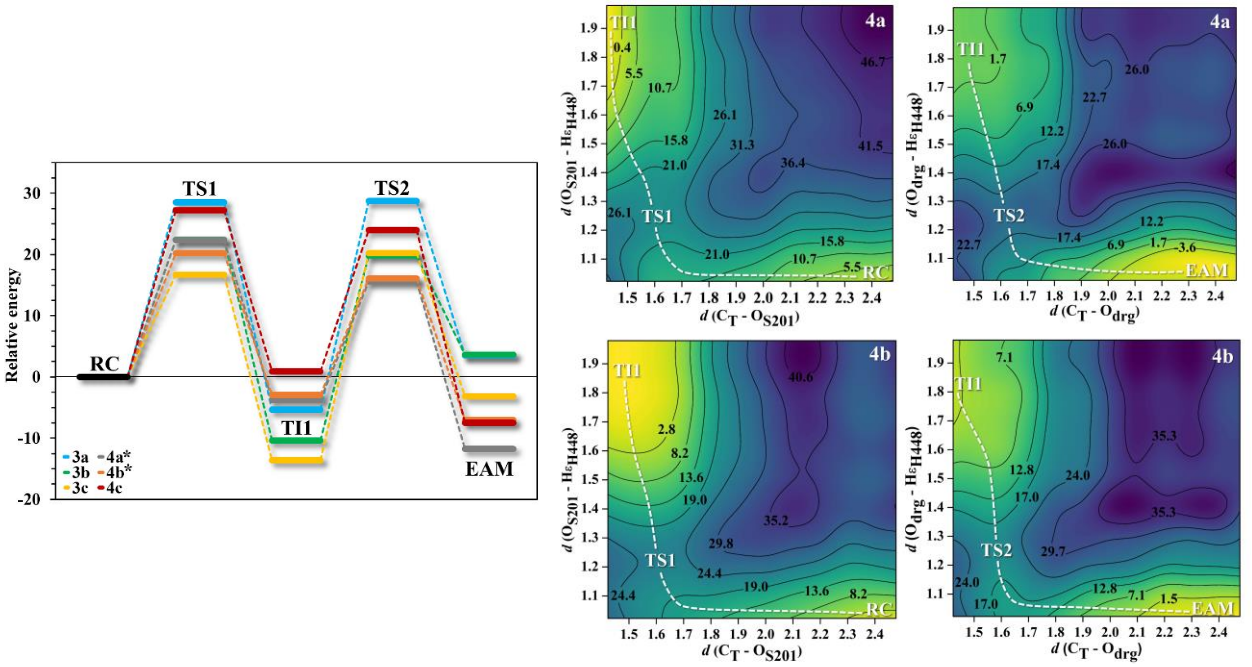

Figure S4. Free energy profiles of PDCs **3a-c** and **4b-c** (**left**) and FEL maps of **4a** and **4b** (**right**) for the acylation step derived from the PES and *FEL. The energetic values were calculated with B3LYP-D3/6-31++G(d,p)/MM[25,51] and are given in kcal mol^{-1}: $\Delta G^{‡}$ **TS1** (**3a**:28.5; **3b**:22.4; **3c**:16.7; **4a**:22.3*; **4b**:20.2*; **4c**:27.2) and $\Delta G^{‡}$ **TS2** (**3a**:34.1; **3b**:30.2; **3c**:33.8; **4a**:19.4*; **4b**:19.1*; **4c**:23.1).

In the functionalized benzylic derivatives (**4a-c**, Figures 6D, 6F, and S7A), the substrate C_T atom and the serine hydroxyl are more than 2.8 Å apart, whereas the S201-H448 hydrogen bond maintains regular in the three moieties (1.8-2.3 Å, Figures 6D, 6F, and S7A).

Energetically, the **TI1** structures of **4a-b** are located at 3.8 and 3.0 kcal mol^{-1} below, while the **4c** is 0.9 kcal mol^{-1} above the **RC** (Figures S5). We observed a lower hydrogen bond towards the O_{drg} in **4a** and **4c** (1.9 Å *versus* 2.1 Å, and 2.4 Å *versus* 3.4 Å, Figures 6E and S7B, respectively), in opposition to **4b,** where a lower distance to O_{S201} is observed (2.4 Å *versus* 1.7 Å, Figure 6D).

The **TS2** barrier amount to more than 30.2 kcal mol^{-1} and the hydrolysis of these conjugates is expected to take too long to occur. However, for PDCs **4a-b**, we have calculated energy barriers below our threshold and submitted them to further characterization. Small deviations were observed between PES and FEL for **4a-b**. For **4a**, these deviations amounted to only 0.1-0.2 kcal mol^{-1} and for **4b,** in particular the **TS2**, this barrier increased by 3.0 kcal mol^{-1}. Nonetheless, derivatives **4a-b** have rate-limiting steps below 22.3 kcal mol^{-1} (Figure S5) and can be classified as candidates for hydrolysis by the hCE2.

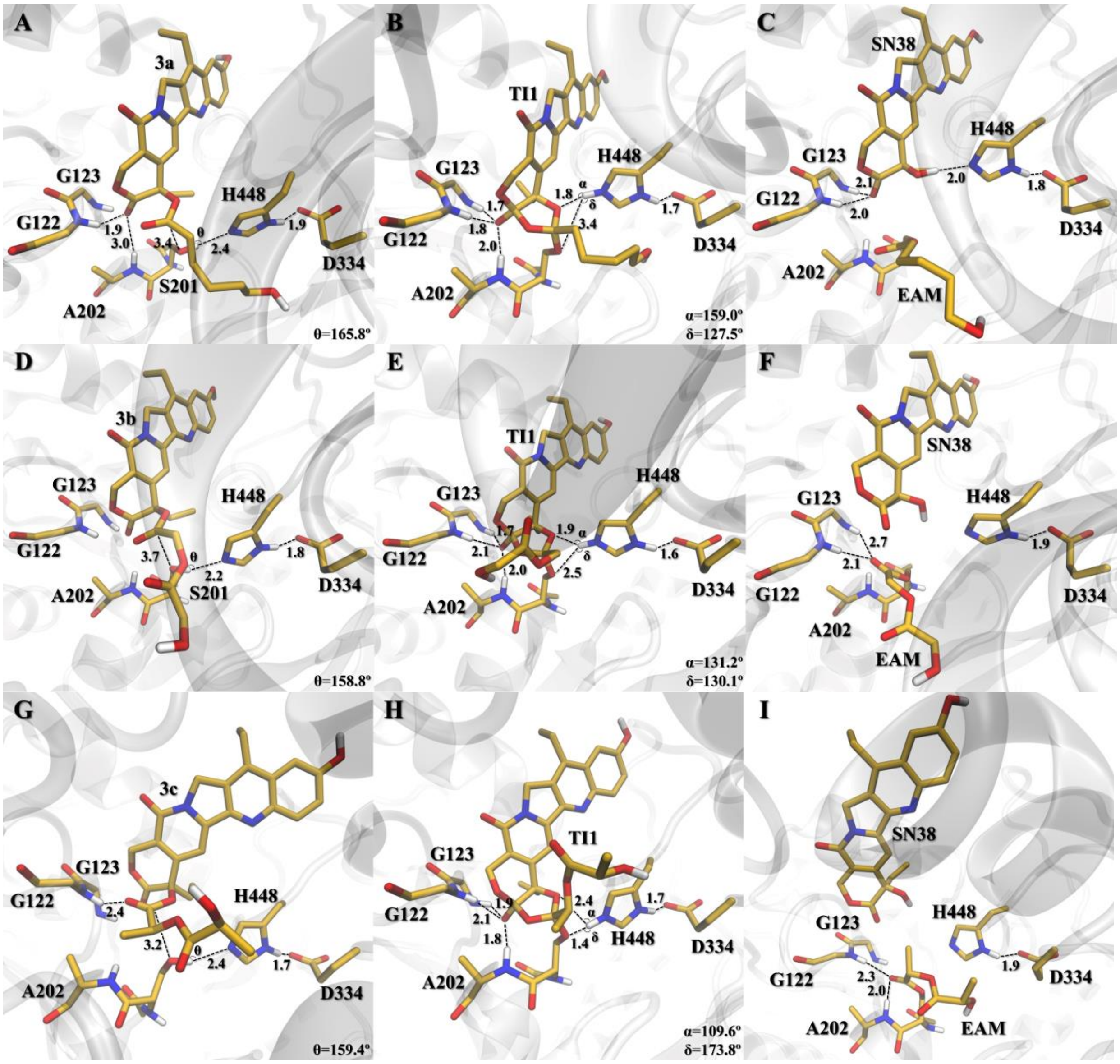

Figure S5. Active site pocket reference structures of the lowest energy stationary points **RC**, **TI1,** and **EAM** of **3a** (**A**, **B**, and **C**, respectively), **3b** (**D**, **E**, and **F**, respectively), and **3c** (**G**, **H**, and **I**, respectively), where key distances are given in Å.

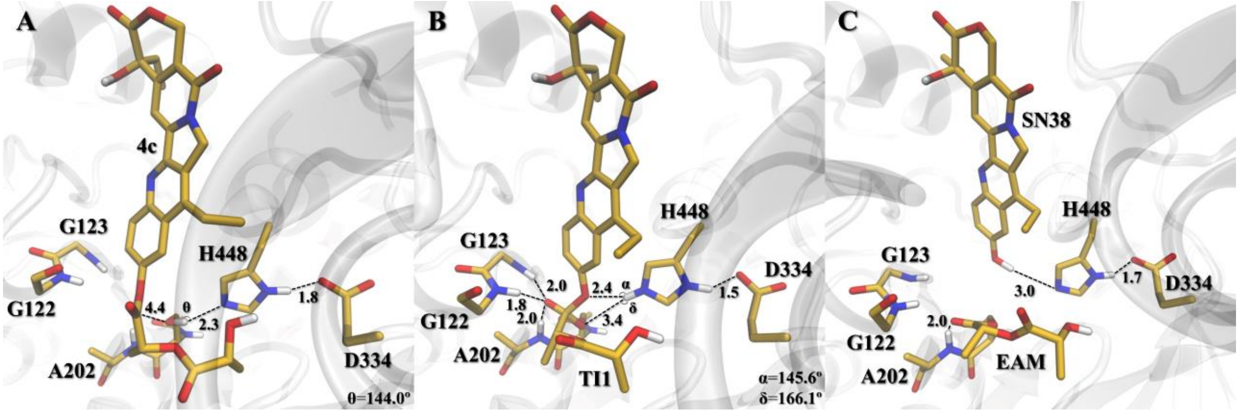

Figure S6. Active site pocket reference structures of the lowest energy stationary points **RC**, **TI1**, and **EAM** of **4c** (**A**, **B**, and **C**, respectively), where key distances are given in Å.

Doxorubicin-based conjugates

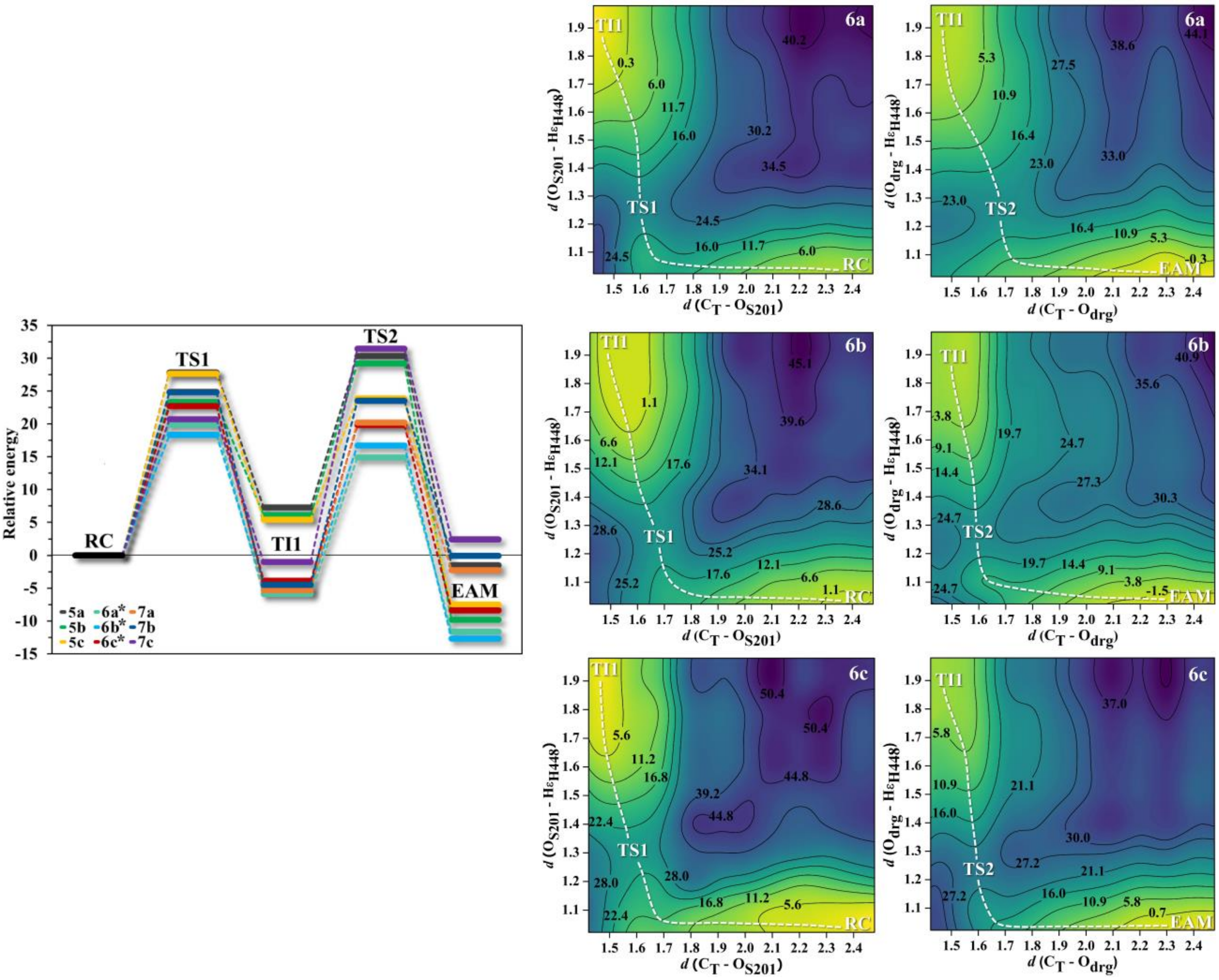

Figure S7. Free energy profiles of PDCs **5a-c**, **6a-c**, and **7b-c** (**left**) and FEL maps of **6a**, **6b**, and **6c** (**right**) for the acylation step derived from the PES and *FEL. The energetic values were calculated with B3LYP-D3/6-31++G(d,p)/MM[25,51] and are given in kcal mol^{-1}: $\Delta G^{\ddagger}$ **TS1** (**5a**:27.8; **5b**:23.3; **5c**:27.6; **6a**:19.9*; **6b**:18.3*; **6c**:22.7*; **7a**:24.9; **7b**:24.8; **7c**:20.7) and $\Delta G^{\ddagger}$ **TS2** (**5a**:30.3; **5b**:29.2; **5c**:18.5; **6a**:20.8*; **6b**:21.6*; **6c**:23.7*; **7a**:25.5; **7b**:28.0; **7c**:32.4).

The first tested doxorubicin derivatives (**5a-c**, Figure 3) are under 3.0 Å from the S201 hydroxyl oxygen in their **RC** complex (Figures S9A, S9D, and S9G). The substrates' approximation results in hydrogen bonds with the oxyanion hole residues, namely A202 (in all three cases), G123 (in **5b**), and G122 (in **5c**). The S201 sidechain proton is below 2.0 Å from the $N\varepsilon_{H448}$ atom with angles for the latter bond above 155.0°. After the achievement of **TS1**, the system will eventually generate the **TI1** structure after releasing around 17.0 kcal mol^{-1}, locating itself above the **RC**.

As previously seen for the other drugs, the $H\varepsilon_{H448}$ is much closer to the O_{S210} rather than O_{drg} atom at the **TI1**. Also, the angles describe a better position of the system for the reverse reaction (to **RC**): 101.1° and 116.5° for **5b-c**, respectively (Figures S9E and S9H). In the **5a TI1**, the angle for the forward reaction is higher but near the threshold for a hydrogen bond, as well as the distance between O_{drg}-$H\varepsilon_{H448}$ (138.2°, 2.9 Å, Figure S9B).

For the hydroxyl directly linked to the allylic ring **6a-c** (Figure 3), the **RC** structures have the substrate C_T atom more than 3.0 Å distanced from the O_{S201} and the corresponding hydrogen atom 1.9/1.8 Å from

Nε$_{H448}$. The angles are well aligned for the reaction to **TS1**, ranging from 148.8º to 160.5º (Figures 7A, 7D, and 7G).

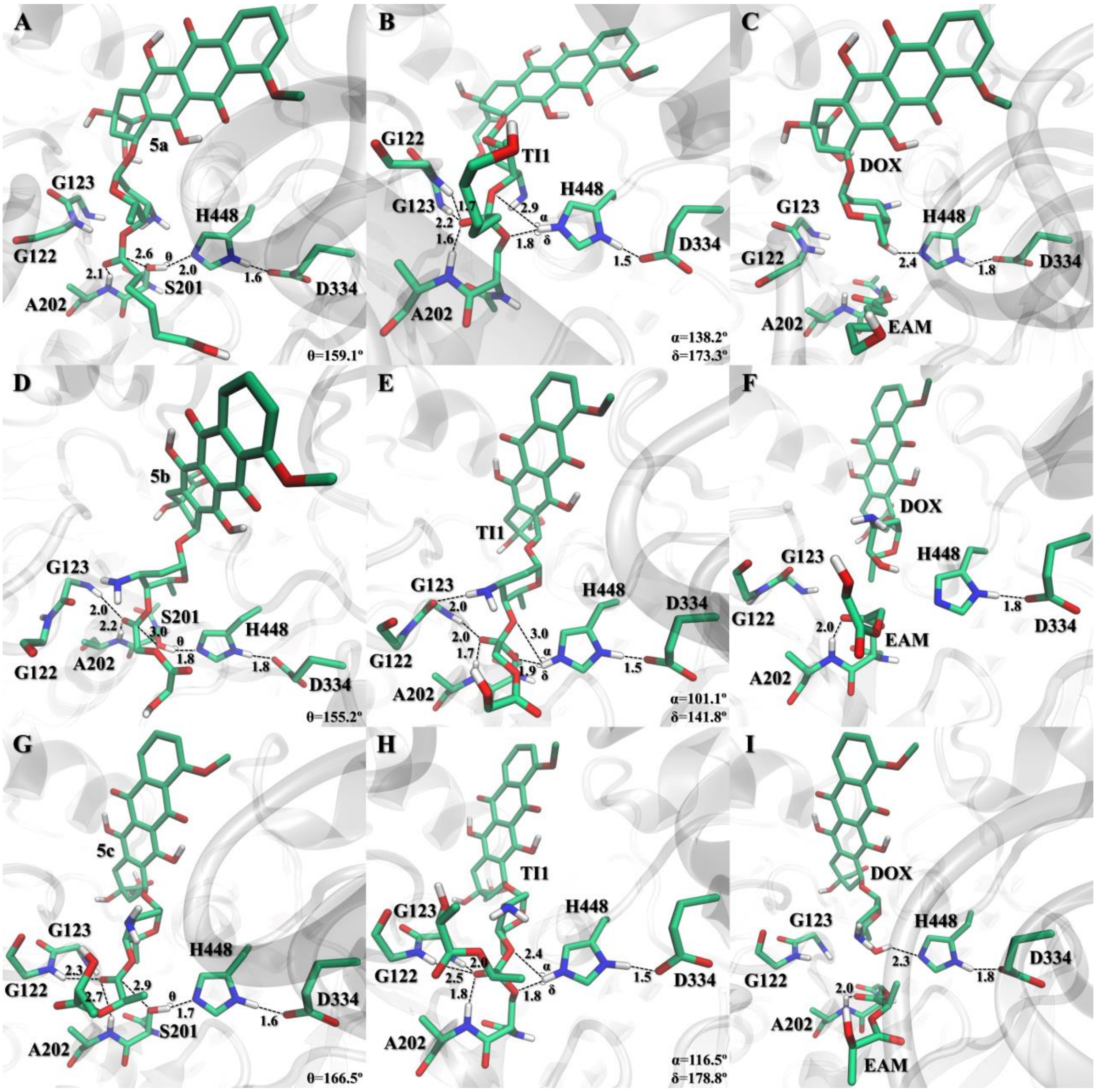

Figure S8. Active site pocket reference structures of the lowest energy stationary points **RC**, **TI1**, and **EAM** of **5a** (**A**, **B**, and **C**, respectively), **5b** (**D**, **E**, and **F**, respectively), and **5c** (**G**, **H**, and **I**, respectively), where key distances are given in Å, and the free doxorubicin drug shortened to **DOX**.

The systems evolve to the **TI1** (Figures 7B, 7E, and 7H), releasing more than 18.8 kcal mol^{-1}, being the formation of the **TI1** exergonic concerning the **RC** (Figure S8). In **6a** and **6c**, the oxyanion hole is stabilizing the negative charge by three hydrogen bonds and the Hε$_{H448}$ is closer to the O$_{drg}$ atom (Figures 7B and 7H). By contrast, in **6b,** the G122 is not interacting with the negatively charged oxygen and the Hε$_{H448}$ atom is equidistant to O$_{S201}$ and O$_{drg}$ (1.9 Å, Figure 7E). The system is better positioned to move the reaction forward, as the angle towards the O$_{drg}$ atom is higher (149.9º *versus* 136.9º, Figure 7E). The released doxorubicin (**EAM**, Figures 7C, 7F, and 7I) is energetically below the **TI1** with more than 4.5 kcal mol^{-1} in the three derivatives (Figure S8).

Comparing the PES and FEL for these systems, we notice small changes in their barriers. For instance, the **TS1** barrier increased around 2.0 kcal mol^{-1} for **6a-b** and 4.1 kcal mol^{-1} in the case of **6c**, which gives us an average barrier of 20.0 kcal mol^{-1} for the **TS1**. Concerning the **TS2**, lower barriers were calculated for **6a-b** (2.5 and 1.2 kcal mol^{-1}, respectively), while in **6c** we observed a small increase of 0.8 kcal mol^{-1}, for an average barrier of 22.0 kcal mol^{-1}. This is also related to energetic changes in the **TI1** structure, which decreased for conjugates **6a-b** and increased for **6c**. Overall, the rate-limiting step for these conjugates (**6a-c**) was predicted to be below 23.7 kcal mol^{-1} (Figure S8) and capable of hydrolysis by the hCE2.

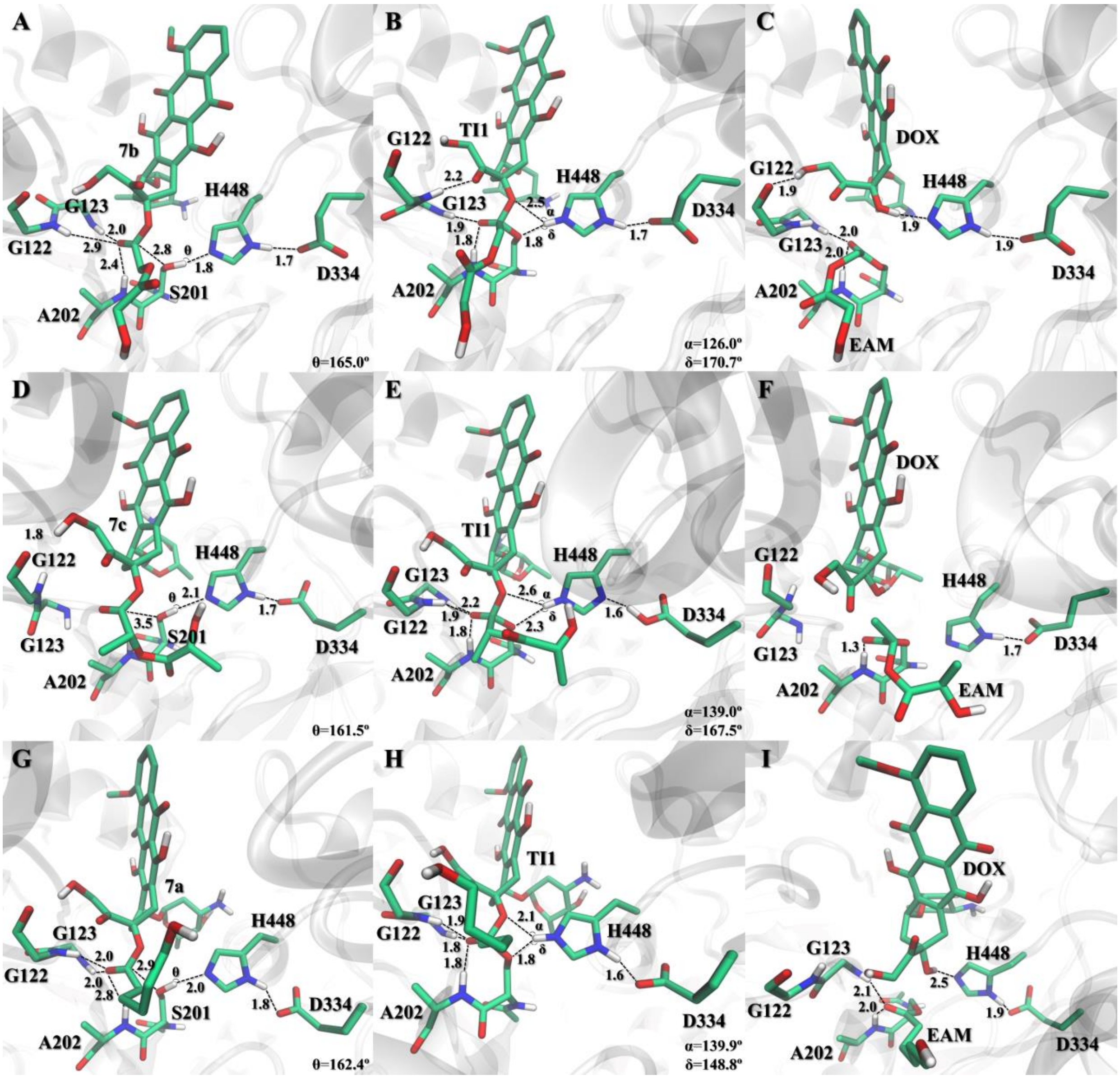

Figure S9. Active site pocket reference structures of the lowest energy stationary points **RC**, **TI1**, and **EAM** of **7a** (**A**, **B**, and **C**, respectively), **7b** (**D**, **E**, and **F**, respectively), and **7c** (**G**, **H**, and **I**, respectively), where key distances are given in Å, and the free doxorubicin drug shortened to **DOX**.

The **7a-c** derivatives were then screened (Figure 3). The **RC** structures have the S201 and the substrate distanced around 2.9 Å for **7a-b,** and 3.5 Å for **7c**. As the substrate enters the active site, the hCE2 oxyanion hole residues start interacting with the oxygen atom that will develop a negative charge (Figures S10A,

S10D, and S10G). In **7c** only the A202 interacts at this step (Figure S10G). The previously observed tendency is also present here: the O_{drg}-$H\varepsilon_{H448}$ distance is larger than the O_{S201}-$H\varepsilon_{H448}$ at the **TI1** structure. Additionally, the angles promote the reverse reaction as they are usually above 167.0º for **7a-b (**Figures S10B and S10E), and a smaller value is observed for **7c** (148.8º, Figure S10H).

Paclitaxel-based conjugates

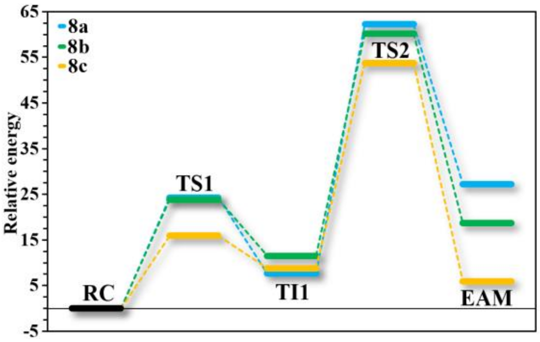

Figure S10. Free energy profiles for the acylation step of PDCs **8a-c** derived from the PES. The energetic values were calculated with B3LYP-D3/6-31++G(d,p)/MM[25,51] and are given in kcal mol^{-1}: $\Delta G^{\ddagger}$ **TS1** (**8a**:24.3; **8b**:23.8; **8c**:15.9) and $\Delta G^{\ddagger}$ **TS2** (**8a**:62.2; **8b**:60.2; **8c**:53.8).

In the **8a-c** (hydroxyl based on the cyclohexane-oxetane fused rings, Figure 3) **RC** structure, the substrate's carbonyl carbon atom, and the O_{S201} are distanced more than 3.0 Å, and the S201 hydroxyl hydrogen is 2.0-2.3 Å from the $N\varepsilon_{H448}$. The **TS1** is located 24.3 and 23.8 kcal mol^{-1} above the **RC** structure for **8a-b**, respectively, and a lower $\Delta G^{\ddagger}$ of 15.9 kcal mol^{-1} was calculated for **5c** (Figure S11). This will lead to the formation of the **TI1** structure, where we observed a pattern concerning the distance between O_{S201} and $H\varepsilon_{H448}$. In the three **8a-c** cases, it is smaller than the O_{drg}-$H\varepsilon_{H448}$ distance, and the angles favor the backward reaction (Figures S12B, S12E, and S12H). Here, the oxyanion hole residues weakly interact with the negatively charged oxygen: the amide bond of A202 is stabilizing this negative charge in the three cases, and the G123 amide hydrogen is only interacting with this substrate atom in **8a** and **8c**, rather than interacting with an oxygen atom other than the negatively charged.

We have also explored the functionalization of the hydroxyl function of the cyclooctane (**9a-c**, Figure 3) and the one located near the benzamide group (**10a-c**, Figure 3), both of which were unable to retrieve a good active site conformation in the cMD simulations. In the case of PDC **9a**, simulations revealed a distortion of the oxyanion hole loop. Concerning PDC **10a**, changes in the oxyanion hole loop structure were also observed. Interactions between the alcohol and amide groups – oriented towards the oxyanion hole loop and residues G122 and G123 – were preventing the interaction of the latter with the substrate's negatively charged atom (Figure 8A). The amide group of G122 is facing and interacting with residues of the loop, while the G123 interacts with the oxygen atom of the substrate amide group. The only active oxyanion hole hydrogen bond is being performed by the A202 amide group (2.5 Å, Figure 8A). aMD simulations for these conjugates were also unable to capture a conformation where the glycine residues from the oxyanion hole are facing and interacting with the negatively charged oxygen atom. Aside from these issues in the negative charge stabilization, this hydroxyl position (Figure 8B) exhibits a stereo effect

executed by the two methyl groups nearby (placed between the O_{drg} and $H\varepsilon_{H448}$), which may block the proton transfer that needs to occur. Although the O_{S201} and $H\varepsilon_{H448}$ atoms are 2.4 Å apart with an angle of 125.9º, the O_{drg} and $H\varepsilon_{H448}$ atoms are much more distanced (3.3 Å) with a worst angle of 88.9º (Figure 8A). This may reflect the VdW forces exercised by the two methyls that are pushing the H448 away from the reactive oxygen atoms. Once these groups are near the O_{drg}, a greater effect can be observed in this oxygen atom, when compared to the more distanced one (O_{S201}).

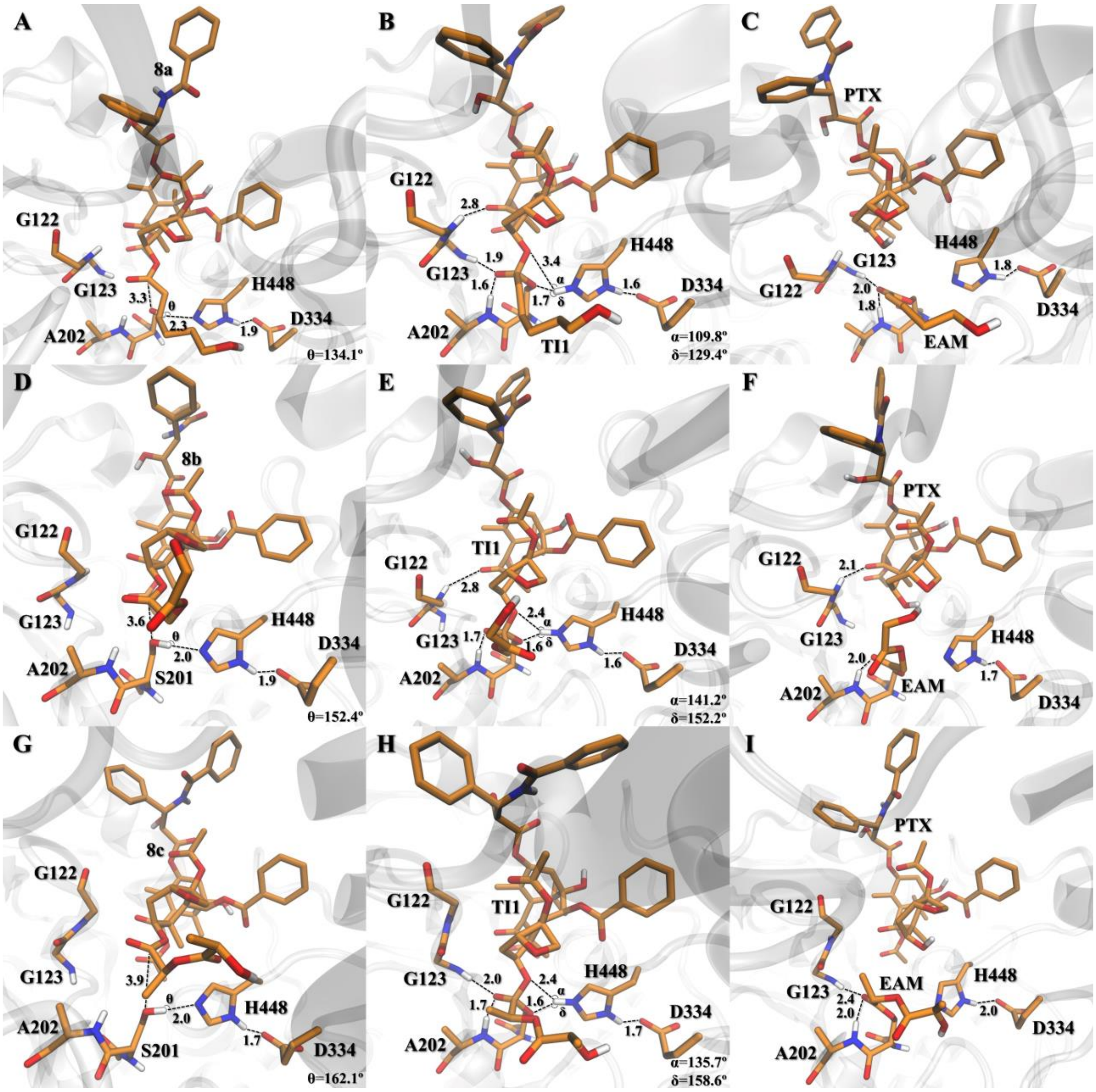

Figure S11. Active site pocket reference structures of the lowest energy stationary points **RC**, **TI1,** and **EAM** of **5a** (**A**, **B**, and **C**, respectively), **5b** (**D**, **E**, and **F**, respectively), and **5c** (**G**, **H**, and **I**, respectively), where key distances are given in Å and the free paclitaxel drug here shortened to **PTX**.

Polymeric carrier release

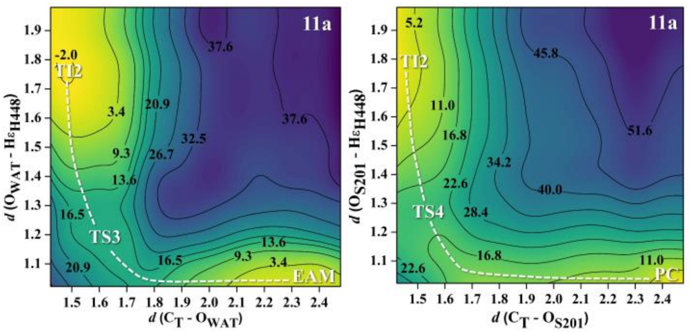

Figure S12. FEL maps of **11a** for the deacylation step. The energetic values were calculated with B3LYP-D3/6-31++G(d,p)/MM[25,51] and are given in kcal mol^{-1}.

Table S1. Selected aMD parameters based on: average total potential energy threshold (α_{dih}), inverse strength boost factor for the total potential energy (α_{pot}), average dihedral energy threshold (E_{dih}), and inverse strength boost factor for the dihedral energy(E_{pot}). The parameter values are given in kcal mol^{-1}.

PDC	α_{dih}	E_{dih}	α_{tot}	E_{tot}
**6a**	373.1	7,676.8	1,683.6	-148,057.6
**7a**	373.1	7,674.9	1,683.8	-164,030.7

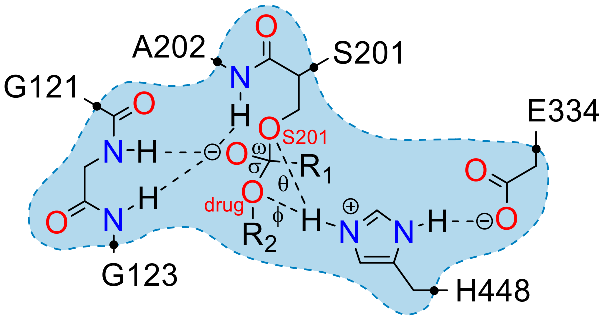

Figure S13. General QM region selected (residues inside the blue area). The link atoms between the QM and MM regions are indicated as black dots. **TI1** R_1=polymer chain, R_2=drug and **TI2** R_1=polymer chain, R_2=H. The complete molecular model defined as QM region of **TI1** and **TI2** is supplied in Figure S14.

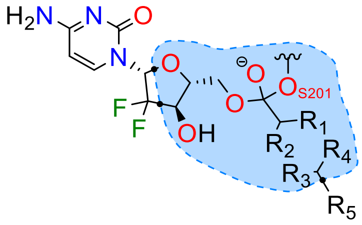

1

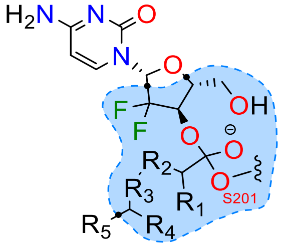

2

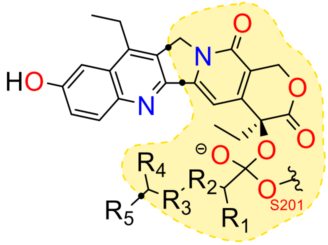

3

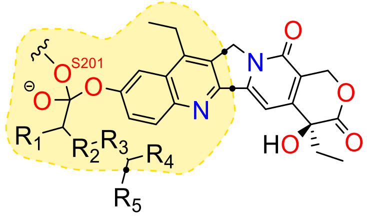

4

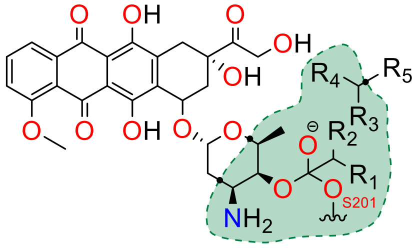

5

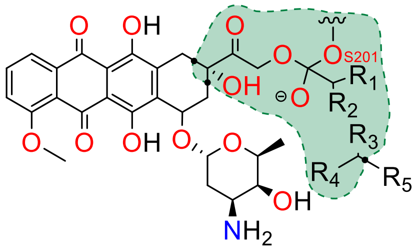

6

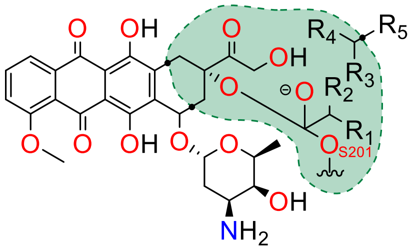

7

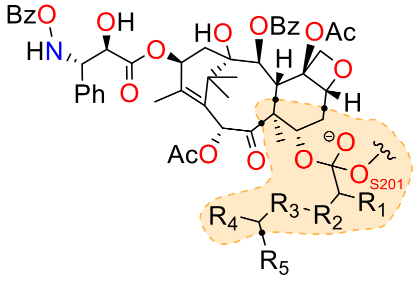

8

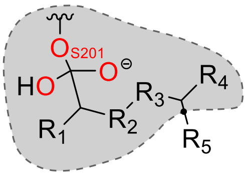

11

Figure S14. Representation of the complete QM region selected for the **TI1** (**1-8**) and **TI2** (**11**). The link atoms between the QM and MM regions are indicated as black dots. **a** (PCL) R_1,R_4=H, R_2,R_3=CH_2, R_5=CH_2OH; **b** (PGA) R_1,R_4=H, R_2=O, R_3=CO, R_5=OH; **c** (PLA) R_1,R_4=CH_3, R_2=O, R_3=CO, R_5=OH.
